# Very high-energy gamma-ray emission beyond 10 TeV from GRB 221009A

**DOI:** 10.1126/sciadv.adj2778

**Published:** 2023-11-15

**Authors:** The LHAASO Collaboration

## Abstract

The highest-energy gamma-rays from gamma-ray bursts (GRBs) have important implications for their radiation mechanism. Here we report the detection of gamma-rays up to 13 teraelectronvolts from the brightest GRB 221009A by the Large High Altitude Air-shower Observatory (LHAASO). The LHAASO-KM2A detector registered more than 140 gamma-rays with energies above 3 teraelectronvolts during 230 to 900 seconds after the trigger. The intrinsic energy spectrum of gamma-rays can be described by a power-law after correcting for extragalactic background light absorption. Such a hard spectrum challenges the synchrotron self-Compton scenario of relativistic electrons for the afterglow emission above several teraelectronvolts. Observations of gamma-rays up to 13 teraelectronvolts from a source with a measured redshift of *z* = 0.151 hints more transparency in intergalactic space than previously expected. Alternatively, one may invoke new physics such as Lorentz invariance violation or an axion origin of very high-energy signals.

## Teaser

LHAASO detected the highest energy gamma-rays, beyond 10 TeV, from the brightest gamma-ray burst GRB 221009A.

## INTRODUCTION

Gamma-ray bursts (GRBs) are sudden explosions of gamma-rays that occur in random directions from cosmological distances. They are the most luminous fireworks in the Universe and are thought to originate from collapsing massive stars and compact star mergers. Prompt flashes and long-lasting afterglows have been thoroughly studied, with thousands of GRBs observed in a wide range of energies from radio waves to megaelectronvolt (MeV) gamma-rays. A fraction of GRBs are also observed with gigaelectronvolt (GeV) gamma-rays. Recently, a handful of GRBs have been observed in teraelectronvolt (TeV) gamma-rays during the afterglow period (*1*–*4*). The synchrotron self-Compton (SSC) process of relativistic electrons in the afterglow has been proposed to explain the origin of the TeV emission (*2*, *5*, *6*). However, challenges (*4*) have been reported for such a scenario. Observation of the highest energy emission from GRBs is important to probe the gamma-ray emission mechanism and particle acceleration processes in GRBs.

The exceptionally bright GRB 221009A [right ascension (RA) = 288.264°, declination (Dec) = 19.768°] was detected by Fermi Gamma-ray Burst Monitor (GBM) on 9 October 2022, at 13:16:59.99 Universal Time (denoted as *T*_0_ hereafter) (*7*), about 1 hour earlier than the Swift trigger (*8*). The GBM light curve consists of two emission episodes: a single isolated peak from *T*_0_ to *T*_0_ + 20 s, followed by a longer, extremely bright, multipulsed emission episode from about *T*_0_ + 220 s to *T*_0_ + 550 s (*7*). It was also detected by the Large Area Telescope (Fermi-LAT) with the highest-energy gamma-ray of 99.3 GeV observed 240 s after the GBM trigger (*9*). This GRB was also clearly observed by many other detectors, such as Insight-HXMT, GECAM-C (*10*), and Konus-Wind (*11*). Follow-up optical observations revealed that the redshift of this GRB is *z* = 0.151 (corresponding to a distance of ∼753 Mpc) (*12*). The estimated isotropic energy release is about 10^55^ ergs (*7*, *10*, *11*), which denotes it as an extremely energetic GRB.

## RESULTS

Large High Altitude Air-shower Observatory (LHAASO) observed GRB 221009A at the highest energy band. LHAASO (*13*) consists of three interconnected detectors located at 4410 m above sea level in Sichuan Province, China. The sub-arrays, a 78,000-m^2^ Water Cherenkov Detector Array (WCDA) and a 1.3-km^2^ Kilometer Squared Array (KM2A), are dedicated to gamma-ray observations with a wide field of view (FOV). Both arrays can monitor the sky for zenith angles less than 50°. GRB 221009A entered the FOV of LHAASO at around *T*_0_ − 20000 s, culminating at a zenith angle of 9.5° at *T*_0_ − 7000 s, and left the FOV at *T*_0_ + 6000 s. For a TeV-detected GRB, the observations cover both the prompt period and the afterglow period. The zenith angles were 28° at *T*_0_ and 31.5° at *T*_0_ + 1000 s, which are favorable positions for LHAASO observation. Within 2000 s after *T*_0_, more than 5000 gamma-rays at >0.5 TeV were detected by WCDA from GRB 221009A with significance above 100 SD (*14*). A previous report has focused on the temporal characteristics of the TeV emission based on WCDA data at lower energies, which revealed the earliest TeV afterglow from GRB 221009A (*15*). The light curve includes a sharp rise at *T*_0_ + 230 s reaching the peak at about *T*_0_ + 245 s and a following smooth decay with a jet break. Here, we focus on the spectral results extending to energies above 10 TeV, as measured by KM2A.

**Fig. 1.**
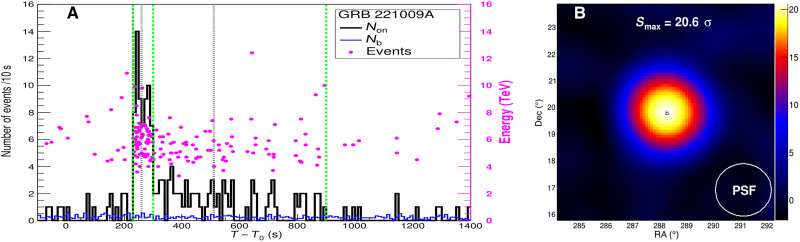
The light curve and significance map of GRB 221009A obtained by KM2A. (**A**) The gamma-ray–count light curve obtained by KM2A with each time bin of 10 s. The black curve indicates the events from the angular cone centered on the GRB, and the blue curve indicates the number of events due to cosmic ray background estimated from 20 similar angular cones at off-source directions with the same zenith angle. The gray dashed lines indicate the peak times of the multipulsed emission observed by GECAM-C (*10*) in the MeV band. The green dashed lines indicate the times of *T*_0_ + 230 s, *T*_0_ + 300 s, and *T*_0_ + 900 s. The pink points indicate the energy marked by the right label and the arrival time of each event. The energies of each event were reconstructed assuming the spectra shown in Fig. 2B. (**B**) The significance map around GRB 221009A as observed by KM2A. The plus sign and corresponding length denote the position and error determined by KM2A. The black circle denotes the position of the GRB reported by Fermi-LAT. The white circle shows the size of the PSF that contains 68% of the events.

KM2A is optimized for gamma-rays with energies from 10 TeV to 10 PeV (*16*). It also has sensitivity to detect gamma-rays below 10 TeV with an effective area of 10,000 m^2^ at 4 TeV and 100,000 m^2^ at 7 TeV, which overlaps with WCDA. Below 10 TeV, KM2A can still remove 98% of the cosmic ray background using a “muon-less” content criterion. The angular resolution is about 1° and the energy resolution is around 40%. The count-rate light curve observed by KM2A is shown in the left of Fig. 1. The emission started at *T*_0_ + 230 s with flux peak around *T*_0_ + 245 s. The emission gradually decreased since *T*_0_ + 300 s and faded out after *T*_0_ + 900 s. During the period from *T*_0_ + 230 s to *T*_0_ + 900 s, 142 events with energies above 3 TeV from the direction of GRB 221009A were detected, and the cosmic ray background was estimated to be 16.7. Therefore, the gamma-ray emission was detected with a significance of 20.6 σ. The position of the GRB was estimated to be RA = 288.26° ± 0.07°, Dec = 19.83° ± 0.07° using the KM2A data, which is consistent with that of Fermi-LAT. The significance map can be found in the right of Fig. 1.

**Fig. 2.**
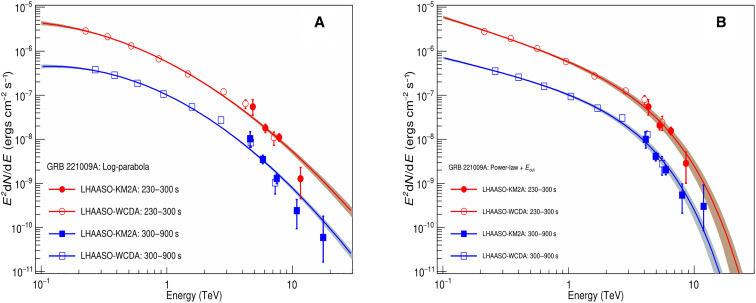
Observed VHE spectra of GRB 221009A by LHAASO for the two intervals. Interval 1 is from *T*_0_ + 230 s to *T*_0_ + 300 s (red points) and interval 2 is from *T*_0_ + 300 s to *T*_0_ + 900 s (blue points). The solid lines indicate the best-fitting results, and the shaded regions indicate the 1-sigma error region. (**A**) The LP function is used to fit the observational data. (**B**) The PLEC function is adopted to fit the observational data.

The KM2A data are divided into two intervals for spectral analysis, according to the light curve shown in Fig. 1, i.e., interval 1 from *T*_0_ + 230 s to *T*_0_ + 300 s and interval 2 from *T*_0_ + 300 s to *T*_0_ + 900 s. We combined the WCDA and KM2A data using a joint forward-folded fit to determine the spectral energy distribution (SED) of GRB 221009A, which is shown in Fig. 2, assuming a log-parabola (denoted as LP) or a power-law with exponential cutoff (denoted as PLEC) function. WCDA covers the energy range from 0.2 to 7 TeV, and KM2A covers the energy range from 3 to 20 TeV. Details about the analysis of KM2A data are presented in Materials and Methods. More details about the analysis of WCDA data can be found elsewhere (*15*). The two measurements are consistent with each other in the overlapping energies from 3 to 7 TeV. Fitting an LP function, the yielded χ^2^/ndf, where ndf is the number of degrees of freedom, are 14.1/9 and 37.3/10, respectively. The probability for the second interval is very low, which indicates that the LP function is not favored. If fitting an PLEC function, then the yielded χ^2^/ndf become much better, with 10.1/9 and 18.3/10 for the two time intervals, respectively. Therefore, the fitting using the PLEC function is better than that using the LP function. The detailed parameters yielded by these two functions for the two intervals are listed in Table 1. The spectrum at interval 2 is slightly harder than that of interval 1. Besides the statistic uncertainty, an addition 7% systematic uncertainty should also exist for the flux according to (*16*). It is worth noting that the precise measurement by LHAASO is the first detection of a cutoff at the high-energy end of a gamma-ray spectrum from a GRB.

**Table 1.** Spectral fitting results for the two time intervals from GRB 221009A. The LP function is $\frac{dN}{dE}=J_{0}{\left(E/1\mathrm{TeV}\right)}^{-a-b.\log(E/1\mathrm{TeV})}$. The PLEC function is $\frac{dN}{dE}=J_{0}{\left(E/1\mathrm{TeV}\right)}^{-a}e^{-E/E_{\mathrm{cut}}}$.

PLE	*J*_0_(10^−8^ TeV^−1^ cm^−2^ s^−1^)	*a*	*E*_cut_ (TeV)	χ^2^/ndf	Probability
230–300 s	51.9 ± 3.3	2.87 ± 0.05	2.62 ± 0.26	10.1/9	0.34
300–900 s	10.5 ± 0.6	2.65 ± 0.05	1.99 ± 0.15	18.3/10	0.050
**LP**			**b**		
230–300 s	35.7 ± 1.3	3.46 ± 0.03	0.58 ± 0.05	14.1/9	0.12
300–900 s	6.37 ± 0.16	3.38 ± 0.02	0.73 ± 0.05	37.3/10	5.0 × 10^−5^
**Saldana-Lopez EBL**					
230–300 s	144 ± 4	2.35 ± 0.03	0 (fixed)	11.0/10	0.36
300–900 s	24.5 ± 0.5	2.26 ± 0.02	0 (fixed)	6.6/11	0.83
**LHAASO-constrained EBL**					
230–300 s	214 ± 6	2.12 ± 0.03	0 (fixed)	5.9/10	0.82
300–900 s	37.8 ± 0.9	2.03 ± 0.02	0.15 ± 0.06	5.5/10	0.86

With the KM2A energy resolution of about 40% at 10 TeV, the reconstruction of energy of an event is nontrivial, in particular for very steep spectra, e.g., in the region of an energy cutoff. To correctly estimate the true energy and corresponding errors of each high-energy event, the probability function of the true energy is constructed for each event, which strongly depends on the assumption of the spectral function. More details can be found in Materials and Methods.

Using the LP function spectra shown in Fig. 2A, eight events with reconstructed energy above 10 TeV were observed during the period from *T*_0_ + 230 s to *T*_0_ + 900 s. The event with the maximum energy is at 17.8_−5.1_^+7.4^ TeV. This energy is similar to that reported in the General Coordinates Network (*14*), which is preliminary estimated using the KM2A alone data assuming a power-law function spectrum. However, if using the PLEC function spectra shown in Fig. 2B, then the energy of the maximum energy event is $12.2_{-2.4}^{+3.5}$ TeV. If the spectral shape is governed by the strongly energy-dependent absorption of gamma-rays in collisions with photons of the extragalactic background light (EBL), which will be discussed later, then the highest energy photon is reconstructed at $12.5_{-2.4}^{+3.2}$ TeV. The energies of each event shown in Fig. 1 were obtained under the PLEC function spectra. The energies of the nine gamma-ray–like events with the highest energies have been estimated under three spectra, i.e., LP, PLEC, and an EBL model. It is worth to note that the LP spectrum is not favored by the data.

There is furthermore a small probability that an event is a misidentified cosmic ray event; the chance probability has been estimated for each event individually and varies for the nine events between 4.5 and 17%. More details can be found in table S3. Gamma-ray events with energy above 100 TeV were searched for during a long period from *T*_0_ to *T*_0_ + 6000 s; however, no event was detected.

### EBL absorption of emission from GRB 221009A

It is known that very high-energy (VHE) gamma-rays emitted from distant astronomical sources are affected by the EBL absorption through photon-photon interaction, inducing a cutoff in the energy spectrum in the form of *e*^−τ(*E*)^ with τ(*E*) being the energy-dependent optical depth. For GRB 221009A at a redshift of *z* = 0.151, the absorption at 1 TeV is modest with a survival fraction ranging from 18 to 21%, while it becomes very heavy at 10 TeV ranging from 0.5 to 0.05%, depending on the EBL models (see fig. S1). The cutoff energies in the two intervals measured by LHAASO are consistent with each other within a 2.1σ error range, which is expected due to EBL absorption. Therefore, the spectra of GRB 221009A measured by LHAASO provide an excellent test of EBL models.

**Fig. 3.**
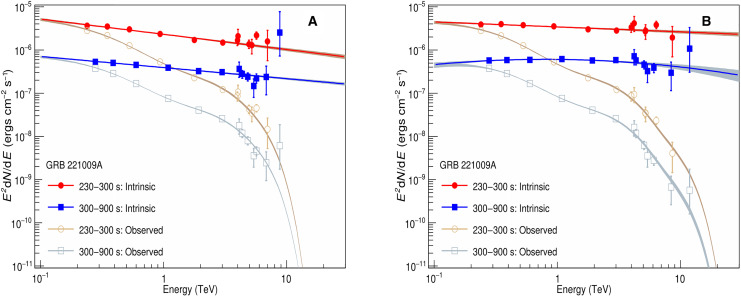
Intrinsic VHE spectra of GRB 221009A corrected for EBL absorption. (**A**) Filled points show the intrinsic spectrum of GRB 221009A corrected for EBL absorption using the model of Saldana-Lopez *et al.* (*17*). The red points are for interval 1 from *T*_0_ + 230 s to *T*_0_ + 300 s, and the blue points are for interval 2 from *T*_0_ + 300 s to *T*_0_ + 900 s. The solid lines indicate the best-fitting results using the power-law function, and the shaded regions indicate the 1-sigma error region. The unfilled points and shaded regions are corresponding observed spectra. (**B**) Filled points show the intrinsic spectrum of GRB 221009A corrected for EBL absorption using the LHAASO-constrained EBL model. The red solid line indicates the best-fitting result for interval 1, which is a power-law function, and the blue solid line indicates the best-fitting result for interval 2, which is a log-parabolic function. The points and shaded regions are similar to those in (A).

To quantitatively test different EBL models, we use a log- parabolic function to describe the intrinsic GRB spectrum and then fit the EBL-attenuated gamma-ray spectrum in the form dN/dE = J_0_*E*^*a*+*b*. log(*E*)^*e*^−τ(*E*)^ to the data. The fitting results are given in the Supplementary Materials. The goodness of fit combining the two intervals is comparable for different EBL models, i.e., Saldana-Lopez *et al.* (*17*), Gilmore *et al.* (*18*), Dominguez *et al.* (*19*), and Finke *et al.* (*20*), as shown in table S3. The intrinsic GRB spectrum obtained using the recent EBL model of Saldana-Lopez *et al.* (*17*) is shown in Fig. 3A. The spectral curvature is insignificant and therefore a simple power-law function is used. The detailed parameters yielded by the fitting for the two intervals are listed in Table 1.

The intrinsic SED of the two intervals follows a single power-law model with an index of −(2.35 ± 0.03) for interval 1 and −(2.26 ± 0.02) for interval 2. For interval 1, the χ^2^/ndf of the fit is 11.0/10, and the maximum deviation is at 5.7 TeV, which deviates from the fit line by 2.3 σ. For interval 2, the χ^2^/ndf of the fit is 6.6/11, while a clear deviation appears at the highest energy point. The measured flux is 11 times higher than the fit line; however, the deviation is not statistically significant due to large flux error (Fig. 3A). This situation is similar to that obtained for the blazar Mrk501 during its huge flare in 1997 (*21*). Although some hypotheses were proposed to produce a nonclassical sharp pile-up spectrum (*22*), a straightforward approach is to reduce the deviation at the highest energy point by decreasing the optical depth at high energy. An empirical method would be to decrease the EBL intensity, the measurement of which still contains large uncertainties due to the heavy contamination caused by foregrounds of different origin, predominantly by the zodiacal (interplanetary dust) light.

**Fig. 4.**
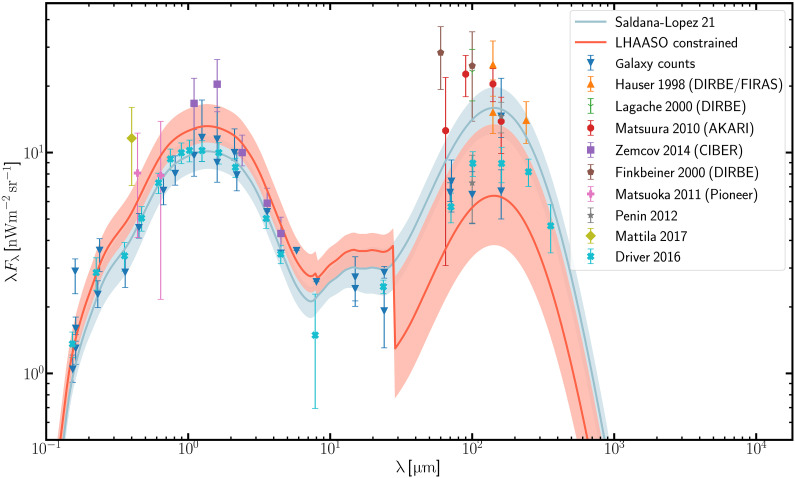
The density of EBL as function of wavelength at *z* = 0. The best-fit EBL from the GRB 221009A measurement is shown with a red line and the corresponding error region in three wavelength bins. The EBL of the Saldana-Lopez *et al.* model (*17*) is shown with a blue line and the corresponding error region. The data points represent direct EBL measurements, taken from (*24*, *35*–*54*). Downward triangles correspond to galaxy counts (*24*), which are taken as the lower limits of EBL.

The absorption of gamma-rays at a specific energy *E* is mainly due to the EBL photons within a narrow interval with Δλ ∼ (1 ± 1/2)λ centered on λ ≈ 1.5(*E*/1TeV)μm (*23*). Therefore, the measured gamma-ray spectra from GRB 221009A can be used to constrain the EBL intensities at different wavelengths. For this purpose, the EBL model of Saldana-Lopez *et al.* (*17*) is independently rescaled at three wavelength ranges, i.e., λ < 8 μm, 8 μm < λ < 28 μm, and λ > 28 μm, which make the dominant contribution to the optical depth for gamma-rays below 5 TeV, between 5 and 10 TeV, and above 10 TeV, respectively. Assuming a log-parabolic intrinsic spectral function by fitting to the LHAASO data, we obtain the rescaled factors for the three EBL ranges as $1.30_{-0.20}^{+0.33}$, $1.20_{-0.20}^{+0.23}$, and $0.40_{-0.16}^{+0.44}$, respectively. The constrained EBL intensity is shown in Fig. 4.

To check the effect of possible absolute energy uncertainty, we have shifted the energy by ±10%. Then, the scale factor of the last EBL bin changes from 0.4 to 0.35 and 0.60, respectively. It should be emphasized that the EBL intensity at large wavelengths is now close to the lower bound set by galaxy count observations (*24*).

The intrinsic spectra using the rescaled best-fit EBL model are shown in Fig. 3B. The detailed parameters yielded by the fitting for the two intervals are listed in Table 1. For interval 1, the intrinsic SED follows a single power-law model with an index of −(2.12 ± 0.03), with a χ^2^/ndf of 5.9/10 yielded by the fitting. For interval 2, the intrinsic SED favors a log-parabolic form with an index of −(2.03 ± 0.02) to (0.15 ± 0.06)log(E/TeV). The corresponding χ^2^/ndf of the fit is 5.5/10. We note that the flux at the highest energy point is much lower now and consistent with the fit spectrum since the EBL absorption is reduced after rescaling EBL for wavelengths λ > 28 μm. Using the LHAASO constrained EBL model and assuming the intrinsic SED shown in Fig. 3B, the energy of the maximum energy event is estimated to be $12.5_{-2.4}^{+3.2}$ TeV.

### EBL absorption corrected light curve from GRB 221009A

**Fig. 5.**
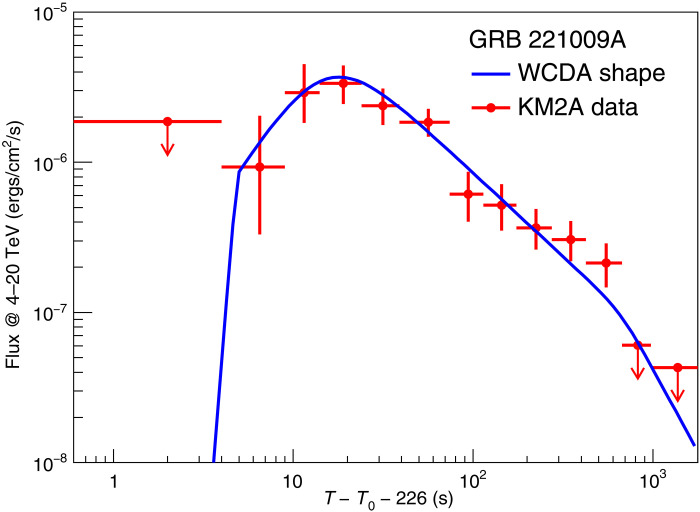
Flux light curve measured by KM2A in the 4- to 20-TeV band for GRB 221009A. Red points indicate the observations, with error bars indicating the ∼1σ statistical uncertainty and upper limits achieved with 95% confidence level. The solid blue curve shows the fitted model adopted in (*15*) to fit WCDA data, which consists of four joint power-laws that describe the four-segment features: rapid rise, slow rise, slow decay, and steep decay. The parameters yielded in (*15*) is directly adopted here while the scaling factor is achieved by fitting the KM2A data points. The χ^2^/ndf of the fit is 5.6/9.

The light curve of GRB 221009A in 0.2 to 5 TeV, as observed by WCDA, is characterized by a rapid rise to a peak, followed by a decay that persists for at least 3000 s after the peak (*15*). The smooth temporal profile suggests an external shock origin for the emission, caused by the interaction of the GRB ejecta with the ambient medium. To compare the temporal variation between WCDA and KM2A emissions, the count rate light curve shown in Fig. 1 is converted to the energy flux light curve assuming the spectra shown in the right of Fig. 3. A direct comparison between the energy flux light curves observed by WCDA and that of KM2A is shown in Fig. 5. The solid line indicates the best-fit four-segment light curve observed by WCDA. Obviously, the light curve observed by KM2A is similar to that of WCDA, indicating that the bulk of KM2A emission should have the same origin as that of the WCDA.

### Limits on new physics using the observation of GRB 221009A

The detection of TeV gamma-rays from a cosmological distance has important implications for possible new physics beyond the standard model (SM) of particle physics. New physics scenarios, such as axions and Lorentz invariance violation (LIV), may affect the process of EBL absorption. To simplify the discussion, we adopt the EBL model of Saldana-Lopez *et al.* (*17*) here.

**Fig. 6.**
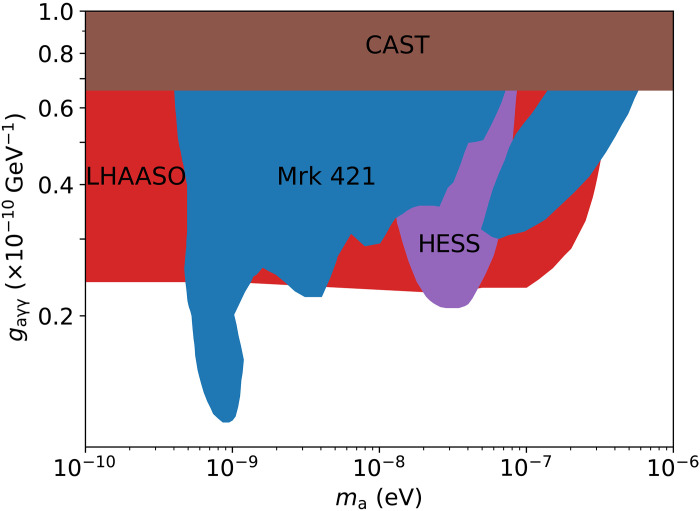
Constraints on the axion–gamma-ray coupling constant by LHAASO observation of the GRB 221009A. It improves the constraint from CAST (*25*) and is comparable with that derived from observations by HESS (*26*) and of Mrk 421 (*27*).

The axion and axion-like particles are hypothetical particles introduced by theoretical models developed beyond the SM. They are among the most attractive cold dark matter candidates. The most distinctive feature of axion is its coupling vertex with two photons, leading to axion–gamma-ray conversion in external magnetic fields for astrophysical detection. The axion–gamma-ray conversion leads to a suppression of EBL absorption of high-energy gamma-rays and higher transparency of space. We still use a log-parabolic form to describe the intrinsic GRB spectrum in this scenario. The χ^2^ of the best fitting for the spectra during the two intervals reduces by only 1.7 compared to that without axion–gamma-ray conversion. Therefore, we give constraints on the parameter space of axion coupling with 95% confidence level. Details of the calculation are given in Materials and Methods. In Fig. 6, the constraint on the axion–gamma-ray coupling constant is shown. It shows that the constraint on the coupling constant by LHAASO observation of GRB 221009A is improved compared with the constraint set by CAST (CERN Axion Solar Telescope) (*25*) and is comparable to that derived from the observation of PKS 2155-304 energy spectrum by High Energy Stereoscopic System (HESS) (*26*) and a long-term monitoring of Mrk 421 by Astrophysical Radiation Ground-based Observatory at YangBaJing (*27*).

Lorentz invariance is a fundamental principle in modern physics. However, some theories that attempt to unify quantum mechanics and general relativity may suggest LIV at the energy scale approaching the Planck scale *M*_Pl_ = 1.22 × 10^28^ eV. This may allow many interesting phenomena that are otherwise forbidden processes (*28*). One consequence is that the threshold of an interaction may be altered. For GRB gamma-rays, if the threshold of interacting with EBL is slightly increased, then the process γγ → *e*^−^*e*^+^ is suppressed, and the universe becomes more transparent. In such a scenario, the detection of gamma rays beyond 10 TeV is easier to understand. However, the reduce of the χ^2^ of the fitting is only 2.6, which is turned to set a constraint on the LIV effect. We derive that for the first-order LIV, the effective energy scale of LIV should be greater than 1.5 *M*_Pl_. This constraint is comparable to that derived by exploring the energy dependence of the propagation speed of gamma-rays from GRB090510 by Fermi-LAT (*29*).

## DISCUSSION

In this work, we report the first detection of gamma-rays beyond 10 TeV from a GRB, GRB 221009A, during the early afterglow period by LHAASO. The measured energy spectrum has a clear cutoff at the high-energy end, which is consistent with the standard picture of gamma-rays propagating and interacting with EBL. The derived intrinsic energy spectrum shows a power-law shape extending beyond 10 TeV, indicating a highly efficient particle acceleration mechanism working within the relativistic shock of the GRB. The LHAASO data prefer a suppression of the EBL flux at mid-infrared wavelengths. These observations also place strong constraints on new physics parameters, such as an axion origin of the signal or LIV. In the following, we will discuss the implication for GRB physics and the possible reasons account for the highest-energy gamma-ray.

### Implication for GRB physics

**Fig. 7.**
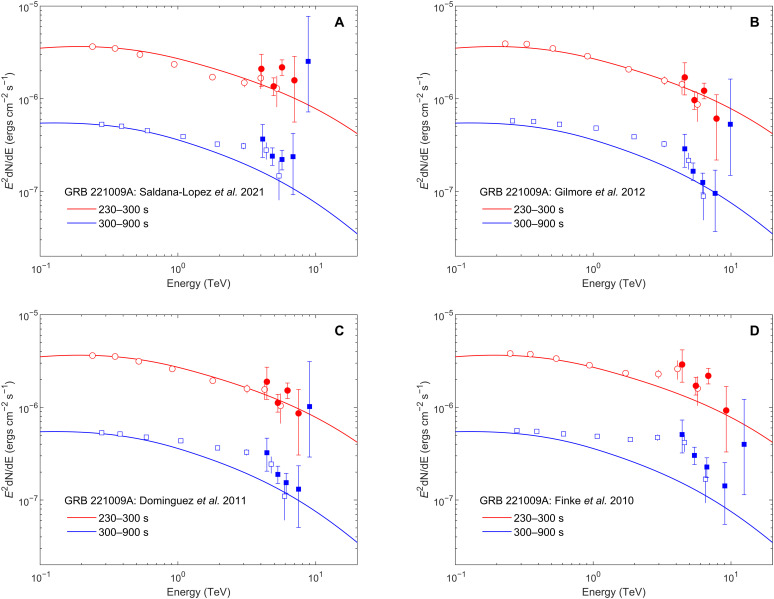
Comparison between the SSC emission model and the LHAASO data for different EBL models. The red points are for the interval from *T*_0_ + 230 s to *T*_0_ + 300 s, while the blue points are for the interval from *T*_0_ + 300 s to *T*_0_ + 900 s. The solid lines are the fitted lines using the SSC emission model (*15*) for corresponding data, respectively. (**A**) The intrinsic spectrum of GRB 221009A corrected for EBL absorption using the Saldana-Lopez *et al.* (*17*) model. (**B**) The intrinsic spectrum of GRB 221009A corrected for EBL absorption using the Gilmore *et al.* (*18*) model. (**C**) The intrinsic spectrum of GRB 221009A corrected for EBL absorption using the Dominguez *et al.* (*19*) model. (**D**) The intrinsic spectrum of GRB 221009A corrected for EBL absorption using the Finke *et al.* (*20*) model.

Figure 3 shows that the intrinsic spectrum of GRB 221009A does not exhibit softening up to at least 10 TeV. For previous TeV afterglows, the SSC process of relativistic electrons has been proposed to produce the TeV emission (*2*, *5*, *6*). However, since the Klein-Nishina effect becomes increasingly notable at higher energies, one would expect a softening of the SSC spectrum toward higher energies (*30*). This can be seen from the comparison between the model that explains the WCDA data (*15*) and the data of KM2A of GRB 221009A (see Fig. 7). This may be similar to the challenge in explaining the TeV data of GRB 190829A with the SSC scenario (*4*). Such a discrepancy would suggest more complicated processes occurring during the early afterglow phase.

One possible solution to this discrepancy for GRB 221009A is to assume that an additional hard spectral component emerges at the highest energy end. In the “standard” paradigm of an external shock, two types of shocks are formed when the ultrarelativistic ejecta are decelerated by the swept-up ambient medium: A forward shock propagating into the external medium that produces the afterglow emission and a reverse shock propagating back into the ejecta shell. In the external shocks, both electrons and protons are accelerated. Proton synchrotron emission from accelerated ultrahigh-energy cosmic rays (UHECRs) in the external reverse shock has been suggested to produce a hard spectral component in GRB 221009A (*31*). The magnetic field in the reverse shock could be larger than that of the forward shock, so protons could be accelerated to ultra-high energies by the reverse shock and thus produce synchrotron emission above 10 TeV (*31*). For a proton spectrum *dN_p_*/*dE* ∼ *E*^−2^, the spectral index of the proton synchrotron emission would be −1.5, much harder than the SSC emission. In addition, the intergalactic electromagnetic cascade due to the propagation of UHECRs (*32*) can also produce a hard spectral component at the highest energy, because >10 TeV gamma-rays could be produced by UHECRs that have propagated to a nearby distance to us. To explain the observed flux of >10 TeV gamma-rays in the hadronic scenario, both models require efficient production of UHECRs from GRBs (*31*, *32*).

An alternative explanation could be to invoke an additional hard leptonic component. This could be realized in the multizone models where the magnetic field is inhomogeneous throughout the emitting volume (*33*). Synchrotron photons from the strong magnetic field zone provide the dominant target for cooling of the electrons in the weak magnetic field zone, which could result in a formation of a hard electron distribution due to the Klein-Nishina scattering effect. A hard electron spectral component could also be formed by the hydrodynamical turbulence that is excited in the GRB forward shock and stochastically accelerates protons and electrons (*34*). The stochastic acceleration can yield a hard electron spectrum with *p* < 2, which has also been discussed as a possible electron acceleration mechanism in active galactic nucleus jets.

### Possible reasons account for the highest-energy gamma-ray event

The detection of a gamma-ray event beyond 10 TeV from a source at a cosmological distance of *z* = 0.151 seems unlikely, considering the heavy absorption by EBL. As shown above, it may hint a suppression of the EBL intensity at large wavelengths. Several possible reasons may account for the observation.

The first possibility is that the highest gamma-ray event is actually a cosmic ray background event with unusually low muon content. The gamma-ray and cosmic ray events recorded by LHAASO are mainly discriminated by the muon content in the air shower. Therefore, a less muonic cosmic ray event could mimic a gamma-ray event. The chance of misidentification has been estimated to be 4.5%, taking into account the arrival time, direction, and the muon content. The second possibility is that this event is actually a lower-energy gamma-ray but is reconstructed to above 10 TeV because of the poor energy resolution. If the intrinsic SED follows the fitting line shown in Fig. 3A and taking the EBL model of Saldana-Lopez *et al.* (*17*), then the expected number of events beyond the highest energy during the interval from *T*_0_ + 300 s to *T*_0_ + 900 s is 0.009, which will be enlarged to 0.015 if using the interval from *T*_0_ + 230 s to *T*_0_ + 900 s. The third possibility is that the absorption of EBL is weaker than the current models predict. As shown in Fig. 3B, the situation improves after decreasing the EBL intensity at wavelengths above 28 μm.

Besides these, other interesting possibilities that avoid EBL absorption may be due to some “exotic” physics, such as LIV or an axion origin of the signal. However, detailed discussions about these scenarios will be plagued with uncertainties of EBL models. Simple constraints on these new physics scenarios have been discussed.

## MATERIALS AND METHODS

KM2A is composed of 5216 electromagnetic particle detectors (EDs) and 1188 muon detectors (MDs), which are distributed in an area of 1.3 km^2^. The EDs are designed to detect the gamma-ray/cosmic ray incident showers with determining their directions and energies. The MDs are designed to detect the muon component of showers, which is used to discriminate between gamma-ray and hadron-induced showers. The whole KM2A detector was completed and operational on 19 July 2021, and the duty cycle is about 99%. A trigger is generated when 20 EDs are fired within a 400-ns window, and the trigger rate is about 2.5 kHz. The performance, including angular resolution, energy resolution, and gamma-ray/cosmic ray discrimination power, of KM2A for gamma-rays has been thoroughly tested using the observation of the Crab Nebula (*16*). The following will show some key information and methods for the observation of GRB 221009A with KM2A.

### Effective area of KM2A for gamma-rays

**Fig. 8.**
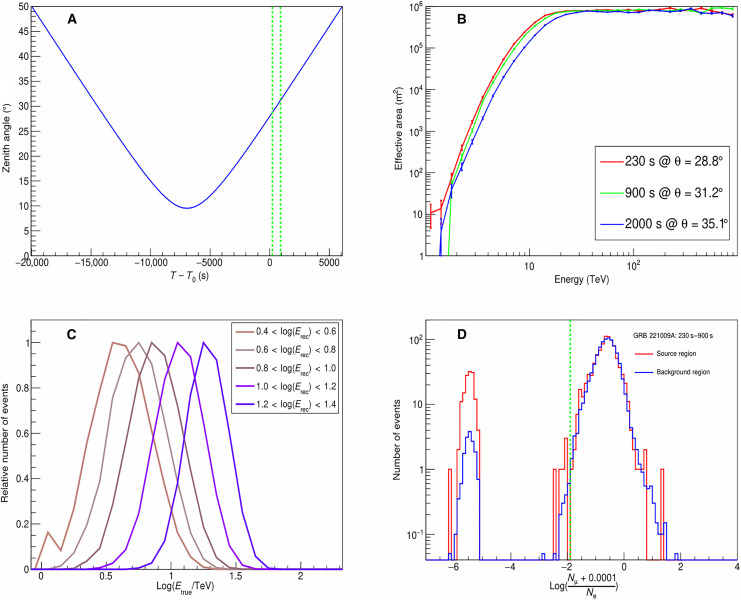
Some key information for the observation of GRB 221009A with KM2A. (**A**) The zenith angle of GRB 221009A within the FOV of LHAASO as a function of the observation time. The dotted green lines mark the times of *T*_0_ + 230 s and *T*_0_ + 900 s, respectively. (**B**) The effective area of KM2A for gamma-rays as a function of the gamma-ray energy at three zenith angles, 28.8°, 31.2°, and 35.1°, corresponding to the observation times *T*_0_ + 230 s, T_0_ + 900 s, *T*_0_ + 2000 s, respectively. (**C**) The distributions of simulated gamma-ray primary true energy (E_true_) in each reconstructed energy (E_rec_) bin in the energy range from 2.5 to 25 TeV. A power-law spectrum with an index of −4.7 is used to derive the *E*_rec_. The number of events has been rescaled to make the peak value 1. The bumpy structure shown in the low-energy tail of the lowest-energy bin is mainly due to statistical fluctuation. (**D**) The distribution of the ratio *R* = log[(*N*_μ_ + 0.0001)/*N*_e_], where *N*_μ_ is the number of muons measured by MDs and *N_e_* is the number of electromagnetic particles measured by EDs. The peak between −5 and −6 are events with *N*_μ_ = 0, i.e., log(0.0001/*N*_e_), smeared out due to the variation of *N*_e_. The red curve represents the events from the GRB source region, while the blue curve represents the events from the 20 off-source regions, with a weight of 1/20 used for each event. The dotted green line marks the position of *R* = −1.9.

The LHAASO observatory arrays can monitor the overhead sky with a zenith angle less than 50°. The zenith angle of GRB 221009A as a function of time is shown in Fig. 8A. The duty cycle of KM2A was 100% during this period. The zenith angles were 28.8° at *T*_0_ + 230 s, 31.2° at *T*_0_ + 900 s, and 35.1° at *T*_0_ + 2000 s. The effective area of KM2A for gamma-rays with different energies at these zenith angles is shown in Fig. 8B. At a zenith angle of 31.2°, the effective area is 10,000 m^2^ at 4 TeV and 100,000 m^2^ at 7 TeV, reaching a roughly constant value of 900,000 m^2^ around 20 TeV. For comparison, the detector area of WCDA is 78,000 m^2^.

### Energy reconstruction for SED measurement

In the normal KM2A data analysis pipeline, the particle density at 50 m (denoted as ρ_50_) from the shower core location is used to evaluate the gamma-ray energy. For a certain zenith angle θ and a certain ρ_50_ value, the probability distribution of the true energy can be achieved using the Bayes theorem

$$P[E|(\rho_{50},\theta )]=\frac{f(E)A_{\mathrm{eff}}(E,\theta )P[\rho_{50}|(E,\theta )]}{\int f(E)A_{\mathrm{eff}}(E,\theta )P[\rho_{50}|(E,\theta )]dE}$$ (1)

where *f*(*E*) is the spectral function, *A*_eff_(*E*, θ) is the effective area at true energy *E* and zenith angle θ, and *P*[ρ_50_∣(*E*, θ)] is the probability to measure ρ_50_ for a gamma-ray event with energy *E* and zenith angle θ. Obviously, the distribution of *P*[*E*∣(ρ_50_, θ)] depends on the assumption of *f*(*E*). Usually, the median value of the distribution of *P*[*E*∣(ρ_50_, θ)] is used as the reconstructed energy *E*_rec_ for a gamma-ray event with a value of ρ_50_ at zenith angle θ assuming a power-law spectral function with index of −3.0 (*16*). Taking into account the derived spectrum for GRB 221009A using the KM2A data, the relation function between ρ_50_ and *E*_rec_ was renewed, assuming a power-law energy spectrum with an index of α = −4.7. This updating will significantly reduce the value of *E*_rec_. In this work, events with reconstructed energy (*E*_rec_) above 2.5 TeV are divided into five bins per decade. The distributions of primary true energy (*E*_true_) of the simulated gamma-ray events in each reconstructed energy bin are shown in Fig. 8C, which roughly presents the energy resolution of KM2A at energies below 25 TeV.

### Gamma-ray and cosmic ray background discrimination

Most of the events recorded by KM2A are cosmic ray–induced showers, which constitute the major background for gamma-ray observations. Considering that gamma-ray–induced showers are muon poor and cosmic ray–induced showers are muon rich, the ratio *R* = log[(*N*_μ_ + 0.0001)/*N*_e_] between the measured muons and electrons is used to discriminate primary gamma-rays from cosmic nuclei. Figure 8D shows the distribution of this ratio for all events from the GRB direction and the background regions. The background regions are 20 off-source regions with the same zenith angle as the GRB. The ratio distribution is about the same for the source region and background region at *R* > −1.9, which is dominated by the cosmic ray background. The source region is clearly higher than the background region at *R* < −1.9, which is mainly due to gamma-ray signals. The events with *R* < −1.9 are used to select gamma-ray–like events to analyze the emission from the GRB in this work. This selection criterion can remove 98% of the cosmic ray background. The survival fraction of gamma-rays is 74% according to Monte Carlo (MC) simulation.

### Spectrum and flux determination

The gamma-ray flux from GRB 221009A is estimated using the number of excess events and the corresponding statistical uncertainty in each energy bin. Combined with the WCDA measurement, the gamma-ray emission from the GRB is assumed to follow an LP spectrum or a PLEC spectrum. The response of the KM2A detector was simulated by tracing the trajectory of the GRB within the FOV of KM2A. The expected energy distribution detected by detector *N*(*E*_rec_) can be achieved

$$N(E_{\mathrm{rec}})=\int \int f(E)A_{\mathrm{eff}}[E,\theta (t)]P\{E_{\mathrm{rec}}|[E,\theta (t)]\}dEdt$$ (2)

where *A*_eff_(*E*, θ) is the effective area at true energy *E* and zenith angle θ, *P*[*E*_rec_∣(*E*, θ)] is the probability to measure *E*_rec_ for an event with energy *E* and zenith angle θ, and θ(*t*) is the zenith angle of the GRB at time *t*. With this energy distribution, the number of signals expected by the MC simulation *N*_MC*_i_*_[*f*(*E*)] using the spectrum *f*(*E*) in each energy bin can be achieved. Then, the best-fit values of the spectrum *f*(*E*) can be obtained using a forward-folded method to minimize a χ^2^ function for all energy bins

$$\chi^{2}=\sum_{i=1}^{n}{\left\{\frac{N_{s_{i}}-N_{MC_{i}}[f(E)]}{\sigma_{Ns_{i}}}\right\}}^{2}$$ (3)

where *N_s_i__* is the number of excess events, and σ*_Ns_i__* is the uncertainty of *N_s_i__* in the *i*th energy bin. *n* denotes the number of energy bins. In this forward-folded method, the biases and energy resolution in the energy assignments shown in Fig. 8 are taken into account. The resulting differential flux has been shown in Fig. 2. The detailed information about these results are listed in table S2. The median energy E_LP_ and E_PLEC_ is the median value of the probability distribution of the true energy *P*[*E*∣(*E*_1_ < *E*_rec_ < *E*_2_)] for a reconstructed energy bin [*E*_1_, *E*_2_] assuming corresponding LP and PLEC spectrum, respectively.

$$P[E|(E_{1}<E_{\mathrm{rec}}<E_{2})]=\frac{\int \int_{E_{1}}^{E_{2}}f(E)A_{\mathrm{eff}}[E,\theta (t)]P\{E_{\mathrm{rec}}|[E,\theta (t)]\}dtdE_{\mathrm{rec}}}{\int \int \int_{E_{1}}^{E_{2}}f(E)A_{\mathrm{eff}}[E,\theta (t)]P\{E_{\mathrm{rec}}|[E,\theta (t)]\}dEdtdE_{\mathrm{rec}}}$$ (4)

The flux at corresponding median energy *E_m_* is achieved using $\frac{N_{S_{i}}}{N_{MC_{i}}[f(E)]}f(E_{m})$.

### Energy reconstruction for the highest energy events

Because of the poor energy resolution of KM2A at the low-energy band, the distribution of *P*[*E*_rec_∣(*E*, θ)] is wide across different *E*_rec_ for a given true energy *E*. Hence, the probability distribution of the true energy *E* for a reconstructed energy *E*_rec_ is also much wide, which can be achieved using the Bayes theorem

$$P[E|(E_{\mathrm{rec}},\theta )]=\frac{f(E)A_{\mathrm{eff}}(E,\theta )P[E_{\mathrm{rec}}|(E,\theta )]}{\int f(E)A_{\mathrm{eff}}(E,\theta )P[E_{\mathrm{rec}}|(E,\theta )]dE}$$ (5)

Obviously, the distribution of *P*[*E*∣(*E*_rec_, θ)] depends on the assumption of the spectral function *f*(*E*). To correctly estimate the true energies and corresponding energy errors of the highest energy events observed by KM2A, the *P*[*E*∣(*E*_rec_, θ)] for each event using different *f*(*E*) is achieved. The median energy and corresponding errors can be achieved by integrating *P*[*E*∣(*E*_rec_, θ)] from 0 TeV to *E*_ξ_ when the corresponding value equal to ξ

$$\xi =\int_{0}^{E_{\xi }}P[E|(E_{\mathrm{rec}},\theta )]dE$$ (6)

The median energy is estimated using ξ = 0.5. The corresponding errors are estimated using ξ = 0.16 and ξ = 0.84, respectively.

## Supplementary Materials

### This PDF file includes:

LHAASO Collaboration Author List Figs. S1 to S3 Tables S1 to S3 References
