# Supplementary material for: Very high-energy gamma-ray emission beyond 10 TeV from GRB 221009A
Source: Sci Adv. 2023 Nov 15;9(46):eadj2778. doi: 10.1126/sciadv.adj2778 (PMC11811911; doi:10.1126/sciadv.adj2778)
Supplement: Supplementary file 1 — LHAASO Collaboration Author List Figs. S1 to S3 Tables S1 to S3 References [file sciadv.adj2778_sm.pdf]

Science Advances
AAAS

Supplementary Materials for

Very high-energy gamma-ray emission beyond 10 TeV from GRB 221009A

The LHAASO Collaboration

Corresponding author: S. Z. Chen, chensz@ihep.ac.cn, X. J. Bi, bixj@ihep.ac.cn;
S. C. Hu, hushicong@ihep.ac.cn; X. Y. Wang xywang@nju.edu.cn

Sci. Adv. **9**, eadj2778 (2023)
DOI: 10.1126/sciadv.adj2778

This PDF file includes:

LHAASO Collaboration Author List
Supplementary Materials
Figs. S1 to S3
Tables S1 to S3
References

LHAASO Collaboration authors and affiliations

Zhen Cao[1,2,3], F. Aharonian[4,5], Q. An[6,7], Axikegu[8], Y.X. Bai[1,3], Y.W. Bao[9], D. Bastieri[10], X.J. Bi[1,2,3], Y.J. Bi[1,3], J.T. Cai[10], Q. Cao[11], W.Y. Cao[7], Zhe Cao[6,7], J. Chang[12], J.F. Chang[1,3,6], A.M. Chen[13], E.S. Chen[1,2,3], Liang Chen[14], Lin Chen[8], Long Chen[8], M.J. Chen[1,3], M.L. Chen[1,3,6], Q.H. Chen[8], S.H. Chen[1,2,3], S.Z. Chen[1,3], T.L. Chen[15], Y. Chen[9], N. Cheng[1,3], Y.D. Cheng[1,3], M.Y. Cui[12], S.W. Cui[11], X.H. Cui[16], Y.D. Cui[17], B.Z. Dai[18], H.L. Dai[1,3,6], Z.G. Dai[7], Danzengluobu[15], D. della Volpe[19], X.Q. Dong[1,2,3], K.K. Duan[12], J.H. Fan[10], Y.Z. Fan[12], J. Fang[18], K. Fang[1,3], C.F. Feng[20], L. Feng[12], S.H. Feng[1,3], X.T. Feng[20], Y.L. Feng[15], S. Gabici[21], B. Gao[1,3], C.D. Gao[20], L.Q. Gao[1,2,3], Q. Gao[15], W. Gao[1,3], W.K. Gao[1,2,3], M.M. Ge[18], L.S. Geng[1,3], G. Giacinti[13], G.H. Gong[22], Q.B. Gou[1,3], M.H. Gu[1,3,6], F.L. Guo[14], X.L. Guo[8], Y.Q. Guo[1,3], Y.Y. Guo[12], Y.A. Han[23], H.H. He[1,2,3], H.N. He[12], J.Y. He[12], X.B. He[17], Y. He[8], M. Heller[19], Y.K. Hor[17], B.W. Hou[1,2,3], C. Hou[1,3], X. Hou[24], H.B. Hu[1,2,3], Q. Hu[7,12], S.C. Hu[1,2,3], D.H. Huang[8], T.Q. Huang[1,3], W.J. Huang[17], X.T. Huang[20], X.Y. Huang[12], Y. Huang[1,2,3], Z.C. Huang[8], X.L. Ji[1,3,6], H.Y. Jia[8], K. Jia[20], K. Jiang[6,7], X.W. Jiang[1,3], Z.J. Jiang[18], M. Jin[8], M.M. Kang[25], T. Ke[1,3], D. Kuleshov[26], K. Kurinov[26], B.B. Li[11], Cheng Li[6,7], Cong Li[1,3], D. Li[1,2,3], F. Li[1,3,6], H.B. Li[1,3], H.C. Li[1,3], H.Y. Li[7,12], J. Li[7,12], Jian Li[7], Jie Li[1,3,6], K. Li[1,3], W.L. Li[20], W.L. Li[13], X.R. Li[1,3], Xin Li[6,7], Y.Z. Li[1,2,3], Zhe Li[1,3], Zhuo Li[27], E.W. Liang[28], Y.F. Liang[28], S.J. Lin[17], B. Liu[7], C. Liu[1,3], D. Liu[20], H. Liu[8], H.D. Liu[23], J. Liu[1,3], J.L. Liu[1,3], J.Y. Liu[1,3], M.Y. Liu[15], R.Y. Liu[9], S.M. Liu[8], W. Liu[1,3], Y. Liu[10], Y.N. Liu[22], R. Lu[18], Q. Luo[17], H.K. Lv[1,3], B.Q. Ma[27], L.L. Ma[1,3], X.H. Ma[1,3], J.R. Mao[24], Z. Min[1,3], W. Mitthumsiri[29], H.J. Mu[23], Y.C. Nan[1,3], A. Neronov[21], Z.W. Ou[17], B.Y. Pang[8], P. Pattarakijwanich[29], Z.Y. Pei[10], M.Y. Qi[1,3], Y.Q. Qi[11], B.Q. Qiao[1,3], J.J. Qin[7], D. Ruffolo[29], A. Sáiz[29], D. Semikoz[21], C.Y. Shao[17], L. Shao[11], O. Shchegolev[26,30], X.D. Sheng[1,3], F.W. Shu[31], H.C. Song[27], Yu.V. Stenkin[26,30], V. Stepanov[26], Y. Su[12], Q.N. Sun[8], X.N. Sun[28], Z.B. Sun[32], P.H.T. Tam[17], Q.W. Tang[31], Z.B. Tang[6,7], W.W. Tian[2,16], C. Wang[32], C.B. Wang[8], G.W. Wang[7], H.G. Wang[10], H.H. Wang[17], J.C. Wang[24], K. Wang[9], L.P. Wang[20], L.Y. Wang[1,3], P.H. Wang[8], R. Wang[20], W. Wang[17], X.G. Wang[28], X.Y. Wang[9], Y. Wang[8], Y.D. Wang[1,3], Y.J. Wang[1,3], Z.H. Wang[25], Z.X. Wang[18], Zhen Wang[13], Zheng Wang[1,3,6], D.M. Wei[12], J.J. Wei[12], Y.J. Wei[1,2,3], T. Wen[18], C.Y. Wu[1,3], H.R. Wu[1,3], S. Wu[1,3], X.F. Wu[12], Y.S. Wu[7], S.Q. Xi[1,3], J. Xia[7,12], J.J. Xia[8], G.M. Xiang[2,14], D.X. Xiao[11], G. Xiao[1,3], G.G. Xin[1,3], Y.L. Xin[8], Y. Xing[14], Z. Xiong[1,2,3], D.L. Xu[13], R.F. Xu[1,2,3], R.X. Xu[27], W.L. Xu[25], L. Xue[20], D.H. Yan[18], J.Z. Yan[12], T. Yan[1,3], C.W. Yang[25], F. Yang[11], F.F. Yang[1,3,6], H.W. Yang[17], J.Y. Yang[17], L.L. Yang[17], M.J. Yang[1,3], R.Z. Yang[7], S.B. Yang[18], Y.H. Yao[25], Z.G. Yao[1,3], Y.M. Ye[22], L.Q. Yin[1,3], N. Yin[20],

X.H. You[1,3], Z.Y. You[1,3], Y.H. Yu[7], Q. Yuan[12], H. Yue[1,2,3], H.D. Zeng[12], T.X. Zeng[1,3,6], W. Zeng[18], M. Zha[1,3], B.B. Zhang[9], F. Zhang[8], H.M. Zhang[9], H.Y. Zhang[1,3], J.L. Zhang[16], L.X. Zhang[10], Li Zhang[18], P.F. Zhang[18], P.P. Zhang[7,12], R. Zhang[7,12], S.B. Zhang[2,16], S.R. Zhang[11], S.S. Zhang[1,3], X. Zhang[9], X.P. Zhang[1,3], Y.F. Zhang[8], Yi Zhang[1,12], Yong Zhang[1,3], B. Zhao[8], J. Zhao[1,3], L. Zhao[6,7], L.Z. Zhao[11], S.P. Zhao[12,20], F. Zheng[32], J.H. Zheng[9], B. Zhou[1,3], H. Zhou[13], J.N. Zhou[14], M. Zhou[31], P. Zhou[9], R. Zhou[25], X.X. Zhou[8], C.G. Zhu[20], F.R. Zhu[8], H. Zhu[16], K.J. Zhu[1,2,3,6], X. Zuo[1,3], (The LHAASO Collaboration)

[1] Key Laboratory of Particle Astrophysics & Experimental Physics Division & Computing Center, Institute of High Energy Physics, Chinese Academy of Sciences, 100049 Beijing, China

[2] University of Chinese Academy of Sciences, 100049 Beijing, China

[3] TIANFU Cosmic Ray Research Center, Chengdu, Sichuan, China

[4] Dublin Institute for Advanced Studies, 31 Fitzwilliam Place, 2 Dublin, Ireland

[5] Max-Planck-Institut for Nuclear Physics, P.O. Box 103980, 69029 Heidelberg, Germany

[6] State Key Laboratory of Particle Detection and Electronics, China

[7] University of Science and Technology of China, 230026 Hefei, Anhui, China

[8] School of Physical Science and Technology & School of Information Science and Technology, Southwest Jiaotong University, 610031 Chengdu, Sichuan, China

[9] School of Astronomy and Space Science, Nanjing University, 210023 Nanjing, Jiangsu, China

[10] Center for Astrophysics, Guangzhou University, 510006 Guangzhou, Guangdong, China

[11] Hebei Normal University, 050024 Shijiazhuang, Hebei, China

[12] Key Laboratory of Dark Matter and Space Astronomy & Key Laboratory of Radio Astronomy, Purple Mountain Observatory, Chinese Academy of Sciences, 210023 Nanjing, Jiangsu, China

[13] Tsung-Dao Lee Institute & School of Physics and Astronomy, Shanghai Jiao Tong University, 200240 Shanghai, China

[14] Key Laboratory for Research in Galaxies and Cosmology, Shanghai Astronomical Observatory, Chinese Academy of Sciences, 200030 Shanghai, China

[15] Key Laboratory of Cosmic Rays (Tibet University), Ministry of Education, 850000 Lhasa, Tibet, China

[16] National Astronomical Observatories, Chinese Academy of Sciences, 100101 Beijing, China

[17] School of Physics and Astronomy (Zhuhai) & School of Physics (Guangzhou) & Sino-French Institute of Nuclear Engineering and Technology (Zhuhai), Sun Yat-sen University, 519000 Zhuhai & 510275 Guangzhou, Guangdong, China

[18] School of Physics and Astronomy, Yunnan University, 650091 Kunming, Yunnan, China
[19] Département de Physique Nucléaire et Corpusculaire, Faculté de Sciences, Université de Genève, 24 Quai Ernest Ansermet, 1211 Geneva, Switzerland
[20] Institute of Frontier and Interdisciplinary Science, Shandong University, 266237 Qingdao, Shandong, China
[21] APC, Université Paris Cité, CNRS/IN2P3, CEA/IRFU, Observatoire de Paris, 119 75205 Paris, France
[22] Department of Engineering Physics, Tsinghua University, 100084 Beijing, China
[23] School of Physics and Microelectronics, Zhengzhou University, 450001 Zhengzhou, Henan, China
[24] Yunnan Observatories, Chinese Academy of Sciences, 650216 Kunming, Yunnan, China
[25] College of Physics, Sichuan University, 610065 Chengdu, Sichuan, China
[26] Institute for Nuclear Research of Russian Academy of Sciences, 117312 Moscow, Russia
[27] School of Physics, Peking University, 100871 Beijing, China
[28] School of Physical Science and Technology, Guangxi University, 530004 Nanning, Guangxi, China
[29] Department of Physics, Faculty of Science, Mahidol University, Bangkok 10400, Thailand
[30] Moscow Institute of Physics and Technology, 141700 Moscow, Russia
[31] Center for Relativistic Astrophysics and High Energy Physics, School of Physics and Materials Science & Institute of Space Science and Technology, Nanchang University, 330031 Nanchang, Jiangxi, China
[32] National Space Science Center, Chinese Academy of Sciences, 100190 Beijing, China

S1 Supplementary Materials

S1.1 LHAASO detector

LHAASO (*13*) consists of three detector arrays: square kilometer array (KM2A), Water Cherenkov Detector Array (WCDA), and Wide-Field-of-view Cherenkov Telescope Array (WFCTA). WFCTA is mainly for cosmic ray physics, while the two particle detector arrays KM2A and WCDA are mainly for gamma-ray physics. When a high-energy extraterrestrial particle, a gamma-ray or cosmic ray, enters Earth's atmosphere, it initiates a cascade consisting of secondary hadrons, muons, leptons, and gamma-rays known as an air shower. The WCDA and KM2A detectors

record different components of these air showers, which are used to reconstruct the type, energy, and arrival direction of the primary particles.

WCDA consists of three water ponds with a total area of 300 m $\times$ 260 m and 3120 detector units. Each detector unit is 5 m $\times$ 5 m and is separated by non-reflecting black plastic curtains and equipped with two upward-facing PMTs on the bottom at the center of the unit. Each pond is filled with purified water up to 4 m above the photo-cathodes of the PMTs. The whole LHAASO-WCDA detector has been operational since March 5th, 2021, and the duty cycle is about 98%. A trigger algorithm was implemented to record air showers by requiring at least 30 PMTs fired among a 12 $\times$ 12 PMT array simultaneously within a window of 250 ns, and the trigger rate is around 35 kHz. The event reconstruction method and the corresponding performance of the array is described elsewhere (*55*).

KM2A is composed of 5216 electromagnetic particle detectors (EDs) and 1188 muon detectors (MDs), which are distributed in an area of 1.3 km^2. Each ED consists of a 1 m^2 plastic scintillator covered by a 0.5 cm thick lead plate and equipped with a 1.5-inch photomultiplier tube (PMT). Each MD consists of a cylindrical water tank, with a diameter of 6.8 m and a height of 1.2 m, and an 8-inch PMT, which is buried under 2.5 m of soil. The MDs are designed to detect the muon component of showers, which is used to discriminate between gamma-ray and hadron-induced showers. The whole KM2A detector was completed and operational on July 19th, 2021, and the duty cycle is about 99%. A trigger is generated when 20 EDs are fired within a 400 ns window, and the trigger rate is about 2.5 kHz. The performance, including angular resolution, energy resolution, and gamma-ray/cosmic-ray discrimination power, of KM2A for gamma-rays has been thoroughly tested using the observation of the Crab Nebula (*16*).

S1.2 The detailed spectral information observed by KM2A

The resulting differential flux has been shown in Figure 2. The detailed information about these results, including the number of events from the source region (N_{on}), the number of background events (N_b), and the differential flux at the median energy (E_{LP} for LP spectrum and E_{PLEC} for PLEC spectrum) of the bin, are listed in Table S1.

S1.3 The highest energy events observed by KM2A

During the period from T_0+230s to T_0+900s, nine events with reconstructed energy above 10 TeV were observed by KM2A, adopting a primary reconstruction as listed in Table S1. Detailed

information about these events is listed in Table S2, including the number of detected secondary electromagnetic particles (N_e), the number of detected muons (N_μ), the incident zenith angle (θ), the distance of the shower core from the nearest edge of the active detector array (D_{edge}), the space angle ($\Delta\psi$) between the event and the direction of the GRB, the arrival time (T_{event}) since T_0, median energy (E_{LP}) and its errors (using the LP spectrum shown in panel A of Figure 2, median energy (E_{PLEC}) and its errors using the PLEC spectrum shown in panel B of Figure 2, and median energy (E_{EBL}) and its errors using the EBL model and spectrum shown in panel B of Figure 3. The chance probability of each event due to background is estimated using the characteristics of each event. Firstly, for each event, the number of background (denoted as b) and signal (denoted as s) events, with arriving time T_{event} during the period from T_0+230s to T_0+900s, ratio less than $log((N_\mu + 0.0001)/N_e)$, space angle with the GRB less than ($\Delta\psi$), and reconstructed energy above E_{rec} of the event, is estimated. Hereafter, the chance probability of the event is calculated using $b/(s + b)$. The chance probability (denoted as P) of each event is also listed in Table S2.

S1.4 SED fitting using different EBL models

The gamma-ray flux from GRB 221009A is estimated using the number of excess events and the corresponding statistical uncertainty in each energy bin. To test different EBL models, such as Saldana-Lopez et al. 2021 (*17*), Gilmore et al. 2012 (*18*), Dominguez et al. 2011 (*19*), and Finke et al. 2010 (*20*), we adopt a log-parabolic form to characterize the intrinsic GRB spectrum (corrected for EBL absorption) and then fit an attenuated model of the form dN/dE = $J_0 E^{a+b.log(E)} e^{-\tau(E)}$ to the data. The intrinsic spectrum using different EBL models is shown in Figure S1. The χ^2/ndf values for different EBL models are listed in Table S3. For the interval from T_0+230s to T_0+300s, the minimum χ^2/ndf is achieved using the Gilmore et al. 2012 model. However, the minimum χ^2/ndf is achieved using the Saldana-Lopez et al. 2021 model for the interval from T_0+300s to T_0+900s. The total χ^2/ndf after summing up the two intervals is comparable in the two cases.

To further test the EBL model, we divided the distribution of EBL adopted in the Saldana-Lopez et al. 2021 (*17*) model into three wavelength ranges, i.e., <8 μm, 8 to 28 μm, and >28 μm. We also adopted a log-parabolic form to characterize the intrinsic GRB spectrum and then fit an attenuated model to the spectral data. We then tuned the scaling factor of each EBL range to minimize the total χ^2/ndf with the sum of the two intervals. According to our fitting result, the best fit factors for the three EBL ranges are $1.30^{+0.33}_{-0.20}$, $1.20^{+0.23}_{-0.20}$, and $0.40^{+0.44}_{-0.16}$, respectively.

The LHAASO constrained EBL model is plotted in Fig. 4. For comparation the Saldana-Lopez EBL model and data points from different EBL measurements are also shown in the figure. The intrinsic SEDs are presented in Figure 3. The corresponding χ^2/ndf values using the best fitting factors for the two intervals are also listed in Table S3.

S1.5 Origin of the ~ 10 TeV gamma-rays

In the work on the WCDA result of GRB 221009A (*15*), the multi-wavelength data, including Swift-XRT, Fermi-LAT, and WCDA data of GRB 221009A, are modeled with the synchrotron plus SSC radiation within the framework of the afterglow emission from external forward shocks. In this model, a GRB jet drives a forward shock expanding into the ambient medium, which accelerates electrons into a power law distribution described by $dN/d\gamma_{\rm e} \propto \gamma_{\rm e}^{-p}$, where $\gamma_{\rm e}$ is the electron Lorentz factor. The modeling takes into account a full Klein-Nishina cross section for the inverse Compton scattering and the internal $\gamma\gamma$ absorption within the shock region.

The comparison between the SSC models used previously for WCDA data (*15*) and at present for KM2A data is given in Figure 7. It can be seen that the SSC emission spectra become increasingly softer at higher energy, thus deviating from the data considerably at the highest energy. This is because both the Klein-Nishina effect and internal $\gamma\gamma$ absorption become stronger at higher energies.

To solve the discrepancy between the SSC model and observed data at the high-energy end, we can assume an additional hard spectral component that becomes dominant at high energies. One possible component is the hadronic emission from the shock-accelerated relativistic protons. It has been proposed that the proton synchrotron emission in the external reverse shock can produce a hadronic component (*31*). The peak energy from proton synchrotron emission can reach ~ 10 TeV if the magnetic field equipartition factor is sufficiently high in the reverse shock (*31*). For a proton spectrum $dN_p/dE \sim E^{-\alpha_p}$, the spectral index of the energy flux (νf_ν) from proton synchrotron emission is $(3-\alpha_p)/2$. With $\alpha_p = 2$, the spectral index of the energy flux is $1/2$, much harder than that of the SSC emission.

Another possibility for the hard spectral component above several TeV is an intergalactic electromagnetic cascade due to the propagation of ultra-high-energy cosmic rays (UHECRs) that are accelerated by internal or external shocks of GRB 221009A (*32, 56, 57*). If these UHECR protons can escape from the source and propagate through the extragalactic medium from their sources to Earth, the interactions lead to the production of secondary cascade parti-

cles. These particles can initiate various energy loss processes for the electromagnetic cascade, such as the inverse-Compton scattering of background photons to higher energies. Because some interactions occur so close to us that the generated TeV gamma-rays do not suffer from significant EBL absorption, the observed cascade radiation could have a hard spectrum (*32*). In this scenario, the extragalactic magnetic field (EGMF) can deflect the UHECRs and cause a time delay. To reconcile with the temporal property of the KM2A emission above a few TeV, which arrived within hundreds of seconds after the GRB trigger, a suitable value of the EGMF is required (*32, 56*). An alternative explanation could be to invoke a new hard leptonic component. This could be realized in the multi-zone models where the magnetic field is inhomogeneous throughout the emitting volume (*33*). Synchrotron photons from the strong magnetic field zone provide the dominant target for the IC cooling of the electrons in the weak magnetic field zone. If the IC cooling is in the Klein-Nishina regime, a hard electron distribution will be formed (*33*). A hard electron spectral component could also be formed by the hydrodynamical turbulence that is excited in the GRB forward shock and stochastically accelerates protons and electrons (*34*). The stochastic acceleration can yield a hard electron spectrum with $p < 2$, though the maximum electron energy depends on the model parameters.

S1.6 Axion-like particle estimation

An axion-like particle (ALP) is a very light pseudoscalar boson with a characteristic coupling to two photons described by the Lagrangian:

$$\mathcal{L}_{a\gamma\gamma} = -\frac{1}{4} g_{a\gamma\gamma} a F_{\mu\nu} \tilde{F}^{\mu\nu} \quad = g_{a\gamma\gamma} a \boldsymbol{E} \cdot \boldsymbol{B} \,, \tag{S1}$$

where a represents the ALP field, $F_{\mu\nu}$ and $\tilde{F}^{\mu\nu}$ are the electromagnetic tensor and its dual, respectively, $\boldsymbol{E}$ and $\boldsymbol{B}$ are the electric and magnetic components, respectively, and $g_{a\gamma\gamma}$ is the coupling constant. According to Eq. (S1), in the presence of external magnetic fields, ALP and gamma-ray conversion $\gamma \leftrightarrow a$ takes place. Therefore, when γ-rays propagate, they oscillate with axions. As a consequence, the absorption of high-energy γ-rays by EBL is weakened, and the optical depth for high-energy γ-rays decreases.

The propagation of γ-rays is described by a Schrödinger-like equation (*58*). The conversion occurs in three different environments, namely, in the source region, in extragalactic space, and in the Milky Way. As a benchmark scenario, we consider the minimal astrophysical environment. In the source region, we assume that the conversion occurs when γ-rays are emitted from

the GRB and propagate in the host galaxy. We assume the transverse magnetic field component to be $0.5\ \mu$G with a coherence length of 10 kpc and an electron density of approximately $0.04\ \mathrm{cm}^{-3}$ (*59*). As the magnetic field in extragalactic space is very weak and has large uncertainties, we ignore the conversion in this region. The magnetic field of the Milky Way is characterized by the regular component of the model in (*60*). The electron density of the Milky Way is described by the NE2001 model (*61*).

Given the circumstances established above and adopting the Saldana-Lopez et al. EBL model (*17*), we solve the propagation equation and obtain the gamma-ray survival probability at Earth. The left panel of Figure S2 shows the gamma-ray survival probability for an axion mass $m_a = 10^{-7}$eV and for different coupling constants. It is shown that the heavy absorption of high energy γ-rays by EBL is greatly alleviated. However, if the coupling constant $g_{a\gamma\gamma}$ is too large, there will be too many high energy gamma-rays detected, and the fitting to data becomes worse, as shown in the right panel of Figure S2. This gives us a constraint on the axion coupling, as shown in Figure 6.

S1.7 Lorentz Invariance Violation

Lorentz Invariance Violation (LIV) modifies the energy-momentum dispersion relation for photons. As a consequence, the threshold energy for photon-photon pair production is changed in the presence of LIV. For the subluminal case of first-order LIV, the energy-momentum conservation yields the modified pair-creation threshold (*62*):

$$\epsilon_{\rm thr} = \frac{m_e^2 c^4}{E} + \frac{1}{8}\left(\frac{E}{E_{\rm LIV}}\right) E\,, \tag{S2}$$

where $\mathrm{E_{LIV}}$ represents the LIV energy scale. From Eq. (S2), it is obvious that the threshold energy is increased, leading to the suppression of the pair production process and more transparency for high energy γ-rays.

The γ-ray survival probability in LIV is calculated in the Saldana-Lopez et al. EBL model for LIV energy scales ranging from 1 $\mathrm{M_{Pl}}$ to 2.8 $\mathrm{M_{Pl}}$, as shown in the left panel of Figure S3. Similar to the axion case, if $E_{\rm LIV}$ is too low, there will be too many high energy gamma-rays, leading to conflicts with observation, as shown in the right panel of Figure S3. This leads us to obtain a lower bound of the LIV energy scale at about 1.5 $\mathrm{M_{Pl}}$.

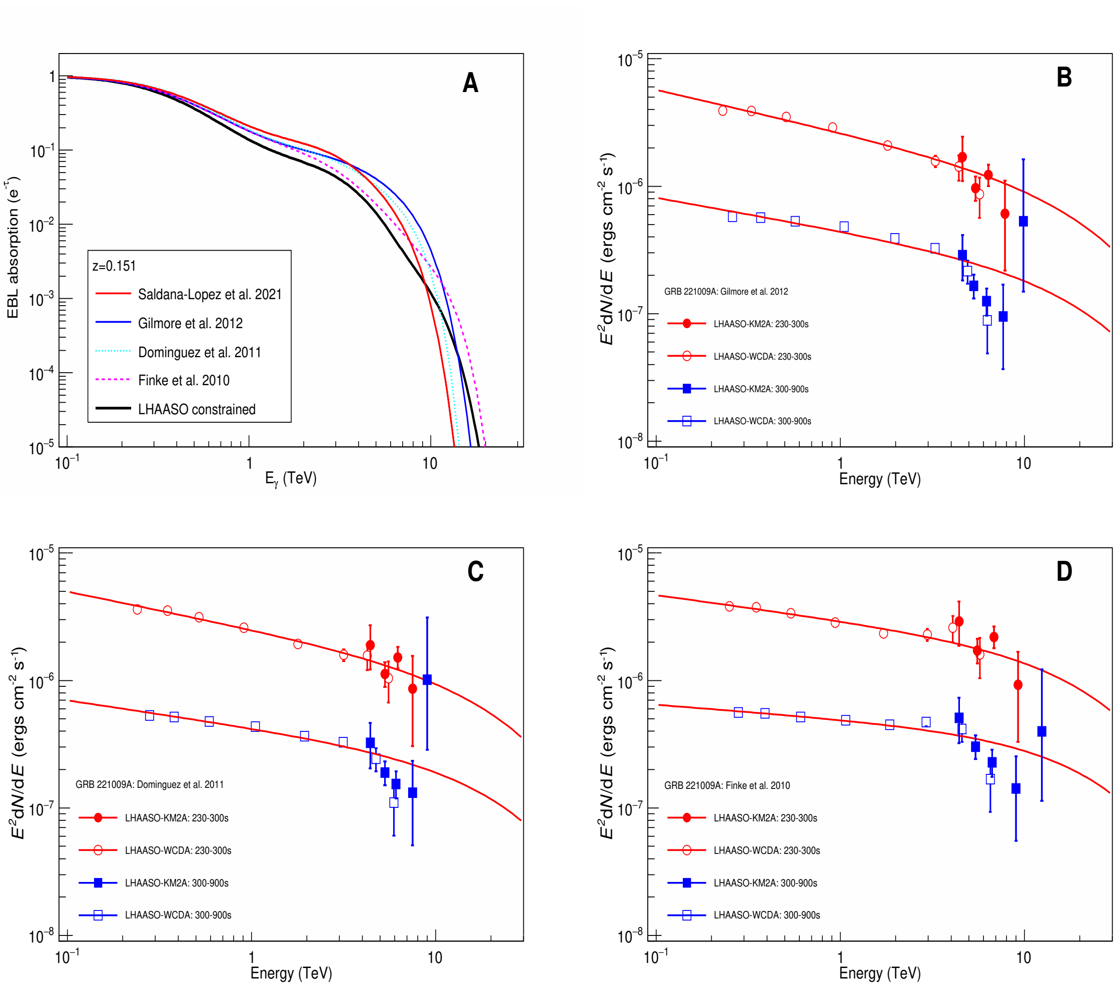

Figure S1: **The EBL absorption and the intrinsic spectrum for gamma-rays from GRB 221009A using different EBL models.** Panel (A) shows the EBL absorption models for VHE gamma-rays from a redshift of z=0.151. Different lines represent different EBL models, i.e., Saldana-Lopez et al. 2021 (*17*), Gilmore et al. 2012 (*18*), Dominguez et al. 2011 (*19*), and Finke et al. 2010 (*20*). Panel (B) shows the intrinsic spectrum of GRB 221009A corrected for EBL absorption using the Gilmore et al. 2012 model. The red points are for the interval from T_0+230s to T_0+300s, while the blue points are for the interval from T_0+300s to T_0+900s. The filled points are obtained using KM2A data, while the unfilled points are obtained using WCDA data. The solid lines are the fitting result using the log-parabolic function. Panels (C) and (D) show the intrinsic spectrum of GRB 221009A corrected for EBL absorption using the EBL models of Dominguez et al. 2011 and Finke et al. 2010, respectively.

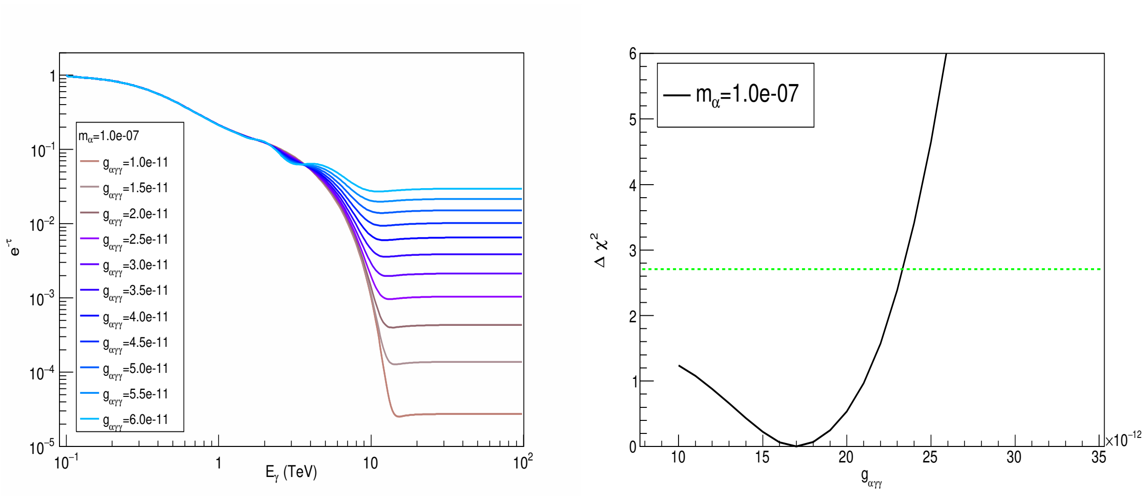

Figure S2: **The EBL absorption and the χ^2 of spectral fitting taking into account the ALP oscillation.** Panel A shows EBL absorption models for very high-energy gamma-rays from a redshift of $z = 0.151$, taking into account the oscillation between gamma-rays and ALPs assuming $m_a = 10^{-7}$ eV and $g_{a\gamma}$=(1 to 6)$\times 10^{-11}$ GeV^{-1}. The EBL model used is Saldana et al. 2021. Panel B shows $\Delta\chi^2$ relative to the minimum that fits the spectral energy distribution data as a function of the ALP $g_{a\gamma}$ for $m_a = 10^{-7}$ eV. The line indicates $\Delta\chi^2 = 2.71$ used to define the upper limit on $g_{a\gamma}$.

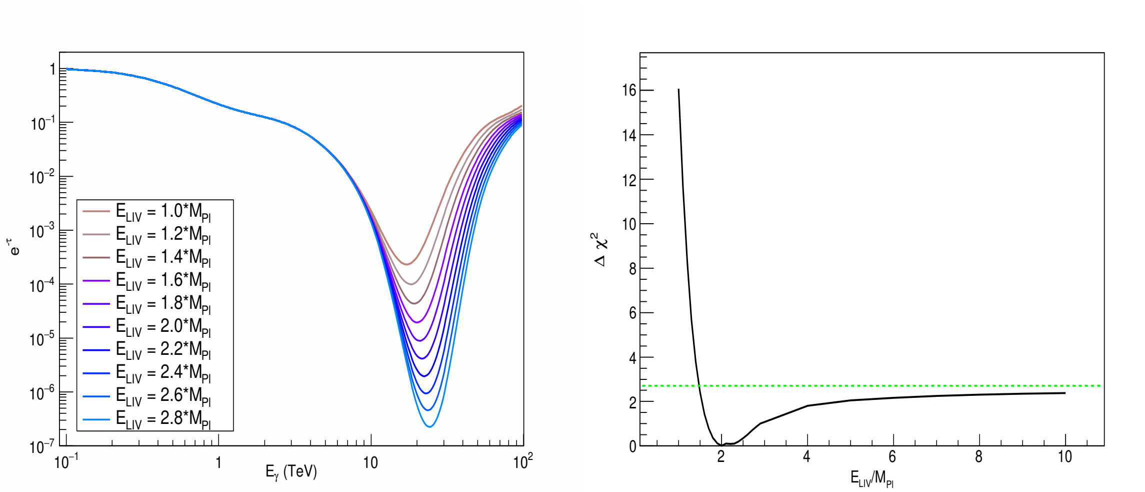

Figure S3: **The EBL absorption and the χ^2 of spectral fitting taking into account the LIV.** (A) EBL absorption models for VHE gamma-rays from a redshift of z=0.151 taking into account the LIV assuming $E_{\rm LIV}$= (1 to 2.8)$\times M_{\rm Pl}$. The EBL model is from Saldana-Lopez et al. 2021. (B) $\Delta\chi^2$ relative to the minimum from fitting the SED data as a function of E_{LIV}. The line indicates $\Delta\chi^2$=2.71 used to achieve the lower limit of $E_{\rm LIV} > 1.5 M_{\rm Pl}$.

Table S1: **The detailed information for the spectral measurement from GRB221009A.** Number of events, background, median energy and corresponding differential flux from GRB 221009A

Time after T_0	$\log(E_{\rm rec}/TeV)$	N_{on}	N_b	E_{LP} (TeV)	Flux (LP/10^{-10}) (ergs cm^{-2} s^{-1})	E_{PLEC} (TeV)	Flux (PLEC/10^{-10}) (ergs cm^{-2} s^{-1})
230-300s	0.4−0.6	8	0.40	4.84	549^{+240}_{-195}	4.32	548^{+239}_{-194}
	0.6−0.8	24	1.20	6.10	179^{+42}_{-37}	5.31	207^{+48}_{-42}
	0.8−1.0	29	1.55	7.85	111^{+23}_{-20}	6.53	156^{+32}_{-28}
	1.0−1.2	3	0.15	11.6	$12.8^{+10.3}_{-8.2}$	8.61	$28.5^{+23.1}_{-18.4}$
	1.2−1.4	0	0	18.8	<11	13.0	<46
	1.4−1.6	0	0	29.2	<15	18.8	<152
300-900s	0.4−0.6	10	1.25	4.62	103^{+45}_{-38}	4.12	100^{+44}_{-37}
	0.6−0.8	34	6.05	5.82	$35.2^{+8.0}_{-7.2}$	4.95	$41.0^{+9.3}_{-8.3}$
	0.8−1.0	28	5.45	7.50	$12.9^{+3.3}_{-3.0}$	5.96	$20.4^{+5.3}_{-4.7}$
	1.0−1.2	5	1.40	10.8	$2.42^{+1.89}_{-1.48}$	8.04	$5.46^{+4.26}_{-3.35}$
	1.2−1.4	1	0.15	17.6	$0.59^{+1.22}_{-0.42}$	11.9	$3.01^{+6.22}_{-2.16}$
	1.4−1.6	0	0	27.2	<2.2	17.2	<29

Table S2: **Detail information of the nine events with the highest energy from GRB 221009A.** The energies for each event are reconstructed using three assuming spectral function.

T_{event}(s)	E_{LP} (TeV)	E_{PLEC} (TeV)	E_{EBL} (TeV)	N_e	N_μ	θ (°)	$\Delta\psi$ (°)	D_{edge} (m)	P (%)
236.6	$12.7^{+6.2}_{-3.8}$	$9.7^{+3.3}_{-2.1}$	$9.8^{+3.1}_{-2.3}$	60.6	0	28.5	0.46	77	7.0
242.5	$10.5^{+5.0}_{-3.2}$	$8.3^{+3.0}_{-2.1}$	$8.4^{+3.2}_{-2.2}$	57.4	0	28.8	0.45	111	10
262.4	$12.6^{+5.5}_{-3.8}$	$9.5^{+3.4}_{-2.3}$	$9.6^{+3.3}_{-2.4}$	57.3	0	28.6	0.53	180	5.7
358.1	$10.0^{+4.8}_{-3.2}$	$7.4^{+3.1}_{-1.8}$	$7.9^{+3.3}_{-2.2}$	46.0	0	28.7	0.54	119	6.0
571.1	$9.4^{+5.1}_{-3.0}$	$7.4^{+2.6}_{-2.5}$	$7.7^{+3.0}_{-2.5}$	45.7	0	29.5	0.52	99	7.8
643.0	$17.8^{+7.4}_{-5.1}$	$12.2^{+3.5}_{-2.4}$	$12.5^{+3.2}_{-2.4}$	81.8	0.3	29.7	0.62	181	4.5
812.4	$11.1^{+5.9}_{-4.3}$	$7.4^{+3.6}_{-2.8}$	$7.6^{+3.9}_{-3.0}$	68.0	0	30.3	0.66	112	11
863.8	$12.9^{+6.1}_{-3.9}$	$9.2^{+3.0}_{-2.8}$	$9.7^{+3.2}_{-3.1}$	100.2	0.8	30.1	1.07	81	17
894.1	$13.6^{+6.1}_{-4.2}$	$9.7^{+3.4}_{-2.5}$	$10.4^{+3.3}_{-3.0}$	60.5	0	31.8	0.83	214	16

Table S3: χ^2/ndf **of the spectral fitting using different EBL models.** The EBL models are Saldana-Lopez et al. 2021 (*17*), Gilmore et al. 2012 (*18*), Dominguez et al. 2011 (*19*),Finke et al. 2010 (*20*), and LHAASO constrained that shown in Figure 4.

EBL model	230-300s	300-900s	total
Saldana-Lopez et al. 2021	11.02/9	5.44/10	16.46/19
Gilmore et al. 2012	3.53/9	15.29/10	18.82/19
Dominguez et al. 2011	4.16/9	11.33/10	15.49/19
Finke et al. 2010	6.12/9	13.51/10	19.63/19
LHAASO constrained	5.93/-	5.49/-	11.42/16

REFERENCES AND NOTES

1. MAGIC Collaboration, V. A. Acciari, S. Ansoldi, L. A. Antonelli, A. Arbet Engels, D. Baack, A. Babić, B. Banerjee, U. Barres de Almeida, J. A. Barrio, J. Becerra González, W. Bednarek, L. Bellizzi, E. Bernardini, A. Berti, J. Besenrieder, W. Bhattacharyya, C. Bigongiari, A. Biland, O. Blanch, G. Bonnoli, Ž. Bošnjak, G. Busetto, A. Carosi, R. Carosi, G. Ceribella, Y. Chai, A. Chilingaryan, S. Cikota, S. M. Colak, U. Colin, E. Colombo, J. L. Contreras, J. Cortina, S. Covino, G. D'Amico, V. D'Elia, P. da Vela, F. Dazzi, A. de Angelis, B. de Lotto, M. Delfino, J. Delgado, D. Depaoli, F. di Pierro, L. di Venere, E. Do Souto Espiñeira, D. Dominis Prester, A. Donini, D. Dorner, M. Doro, D. Elsaesser, V. Fallah Ramazani, A. Fattorini, A. Fernández-Barral, G. Ferrara, D. Fidalgo, L. Foffano, M. V. Fonseca, L. Font, C. Fruck, S. Fukami, S. Gallozzi, R. G. Lopez, M. Garczarczyk, S. Gasparyan, M. Gaug, N. Giglietto, F. Giordano, N. Godinović, D. Green, D. Guberman, D. Hadasch, A. Hahn, J. Herrera, J. Hoang, D. Hrupec, M. Hütten, T. Inada, S. Inoue, K. Ishio, Y. Iwamura, L. Jouvin, D. Kerszberg, H. Kubo, J. Kushida, A. Lamastra, D. Lelas, F. Leone, E. Lindfors, S. Lombardi, F. Longo, M. López, R. López-Coto, A. López-Oramas, S. Loporchio, B. Machado de Oliveira Fraga, C. Maggio, P. Majumdar, M. Makariev, M. Mallamaci, G. Maneva, M. Manganaro, K. Mannheim, L. Maraschi, M. Mariotti, M. Martinez, S. Masuda, D. Mazin, S. Mićanović, D. Miceli, M. Minev, J. M. Miranda, R. Mirzoyan, E. Molina, A. Moralejo, D. Morcuende, V. Moreno, E. Moretti, P. Munar-Adrover, V. Neustroev, C. Nigro, K. Nilsson, D. Ninci, K. Nishijima, K. Noda, L. Nogués, M. Nöthe, S. Nozaki, S. Paiano, J. Palacio, M. Palatiello, D. Paneque, R. Paoletti, J. M. Paredes, P. Peñil, M. Peresano, M. Persic, P. G. Prada Moroni, E. Prandini, I. Puljak, W. Rhode, M. Ribó, J. Rico, C. Righi, A. Rugliancich, L. Saha, N. Sahakyan, T. Saito, S. Sakurai, K. Satalecka, K. Schmidt, T. Schweizer, J. Sitarek, I. Šnidarić, D. Sobczynska, A. Somero, A. Stamerra, D. Strom, M. Strzys, Y. Suda, T. Surić, M. Takahashi, F. Tavecchio, P. Temnikov, T. Terzić, M. Teshima, N. Torres-Albà, L. Tosti, S. Tsujimoto, V. Vagelli, J. van Scherpenberg, G. Vanzo, M. Vazquez Acosta, C. F. Vigorito, V. Vitale, I. Vovk, M. Will, D. Zaric, L. Nava, Teraelectronvolt emission from the γ-ray burst GRB 190114C. *Nature* **575**, 455–458 (2019).

2. MAGIC Collaboration, V. A. Acciari, S. Ansoldi, L. A. Antonelli, A. Arbet Engels, D. Baack, A. Babić, B. Banerjee, U. Barres de Almeida, J. A. Barrio, J. Becerra González, W. Bednarek, L. Bellizzi, E. Bernardini, A. Berti, J. Besenrieder, W. Bhattacharyya, C. Bigongiari, A. Biland, O. Blanch, G. Bonnoli, Ž. Bošnjak, G. Busetto, R. Carosi, G. Ceribella, Y. Chai, A. Chilingaryan, S. Cikota, S. M. Colak, U. Colin, E. Colombo, J. L. Contreras, J. Cortina, S. Covino, V. D'Elia, P. da Vela, F. Dazzi, A.

de Angelis, B. de Lotto, M. Delfino, J. Delgado, D. Depaoli, F. di Pierro, L. di Venere, E. Do Souto Espiñeira, D. D. Prester, A. Donini, D. Dorner, M. Doro, D. Elsaesser, V. Fallah Ramazani, A. Fattorini, G. Ferrara, D. Fidalgo, L. Foffano, M. V. Fonseca, L. Font, C. Fruck, S. Fukami, R. J. García López, M. Garczarczyk, S. Gasparyan, M. Gaug, N. Giglietto, F. Giordano, N. Godinović, D. Green, D. Guberman, D. Hadasch, A. Hahn, J. Herrera, J. Hoang, D. Hrupec, M. Hütten, T. Inada, S. Inoue, K. Ishio, Y. Iwamura, L. Jouvin, D. Kerszberg, H. Kubo, J. Kushida, A. Lamastra, D. Lelas, F. Leone, E. Lindfors, S. Lombardi, F. Longo, M. López, R. López-Coto, A. López-Oramas, S. Loporchio, B. Machado de Oliveira Fraga, C. Maggio, P. Majumdar, M. Makariev, M. Mallamaci, G. Maneva, M. Manganaro, K. Mannheim, L. Maraschi, M. Mariotti, M. Martínez, D. Mazin, S. Mićanović, D. Miceli, M. Minev, J. M. Miranda, R. Mirzoyan, E. Molina, A. Moralejo, D. Morcuende, V. Moreno, E. Moretti, P. Munar-Adrover, V. Neustroev, C. Nigro, K. Nilsson, D. Ninci, K. Nishijima, K. Noda, L. Nogués, S. Nozaki, S. Paiano, M. Palatiello, D. Paneque, R. Paoletti, J. M. Paredes, P. Peñil, M. Peresano, M. Persic, P. G. Prada Moroni, E. Prandini, I. Puljak, W. Rhode, M. Ribó, J. Rico, C. Righi, A. Rugliancich, L. Saha, N. Sahakyan, T. Saito, S. Sakurai, K. Satalecka, K. Schmidt, T. Schweizer, J. Sitarek, I. Šnidarić, D. Sobczynska, A. Somero, A. Stamerra, D. Strom, M. Strzys, Y. Suda, T. Surić, M. Takahashi, F. Tavecchio, P. Temnikov, T. Terzić, M. Teshima, N. Torres-Albà, L. Tosti, V. Vagelli, J. van Scherpenberg, G. Vanzo, M. Vazquez Acosta, C. F. Vigorito, V. Vitale, I. Vovk, M. Will, D. Zarić, L. Nava, P. Veres, P. N. Bhat, M. S. Briggs, W. H. Cleveland, R. Hamburg, C. M. Hui, B. Mailyan, R. D. Preece, O. J. Roberts, A. von Kienlin, C. A. Wilson-Hodge, D. Kocevski, M. Arimoto, D. Tak, K. Asano, M. Axelsson, G. Barbiellini, E. Bissaldi, F. Fana Dirirsa, R. Gill, J. Granot, J. McEnery, N. Omodei, S. Razzaque, F. Piron, J. L. Racusin, D. J. Thompson, S. Campana, M. G. Bernardini, N. P. M. Kuin, M. H. Siegel, S. B. Cenko, P. O'Brien, M. Capalbi, A. Dai, M. de Pasquale, J. Gropp, N. Klingler, J. P. Osborne, M. Perri, R. L. C. Starling, G. Tagliaferri, A. Tohuvavohu, A. Ursi, M. Tavani, M. Cardillo, C. Casentini, G. Piano, Y. Evangelista, F. Verrecchia, C. Pittori, F. Lucarelli, A. Bulgarelli, N. Parmiggiani, G. E. Anderson, J. P. Anderson, G. Bernardi, J. Bolmer, M. D. Caballero-García, I. M. Carrasco, A. Castellón, N. Castro Segura, A. J. Castro-Tirado, S. V. Cherukuri, A. M. Cockeram, P. D'Avanzo, A. di Dato, R. Diretse, R. P. Fender, E. Fernández-García, J. P. U. Fynbo, A. S. Fruchter, J. Greiner, M. Gromadzki, K. E. Heintz, I. Heywood, A. J. van der Horst, Y. D. Hu, C. Inserra, L. Izzo, V. Jaiswal, P. Jakobsson, J. Japelj, E. Kankare, D. A. Kann, C. Kouveliotou, S. Klose, A. J. Levan, X. Y. Li, S. Lotti, K. Maguire, D. B. Malesani, I. Manulis, M. Marongiu, S. Martin, A. Melandri, M. J. Michalowski, J. C. A. Miller-Jones,

K. Misra, A. Moin, K. P. Mooley, S. Nasri, M. Nicholl, A. Noschese, G. Novara, S. B. Pandey, E. Peretti, C. J. Pérez Del Pulgar, M. A. Pérez-Torres, D. A. Perley, L. Piro, F. Ragosta, L. Resmi, R. Ricci, A. Rossi, R. Sánchez-Ramírez, J. Selsing, S. Schulze, S. J. Smartt, I. A. Smith, V. V. Sokolov, J. Stevens, N. R. Tanvir, C. C. Thöne, A. Tiengo, E. Tremou, E. Troja, A. de Ugarte Postigo, A. F. Valeev, S. D. Vergani, M. Wieringa, P. A. Woudt, D. Xu, O. Yaron, D. R. Young, Observation of inverse Compton emission from a long γ-ray burst. *Nature* **575**, 459–463 (2019).

3. H. Abdalla, R. Adam, F. Aharonian, F. Ait Benkhali, E. O. Angüner, M. Arakawa, C. Arcaro, C. Armand, H. Ashkar, M. Backes, V. Barbosa Martins, M. Barnard, Y. Becherini, D. Berge, K. Bernlöhr, E. Bissaldi, R. Blackwell, M. Böttcher, C. Boisson, J. Bolmont, S. Bonnefoy, J. Bregeon, M. Breuhaus, F. Brun, P. Brun, M. Bryan, M. Büchele, T. Bulik, T. Bylund, M. Capasso, S. Caroff, A. Carosi, S. Casanova, M. Cerruti, T. Chand, S. Chandra, A. Chen, S. Colafrancesco, M. Curyło, I. D. Davids, C. Deil, J. Devin, P. de Wilt, L. Dirson, A. Djannati-Atas, A. Dmytriiev, A. Donath, V. Doroshenko, J. Dyks, K. Egberts, G. Emery, J. P. Ernenwein, S. Eschbach, K. Feijen, S. Fegan, A. Fiasson, G. Fontaine, S. Funk, M. Füßling, S. Gabici, Y. A. Gallant, F. Gaté, G. Giavitto, L. Giunti, D. Glawion, J. F. Glicenstein, D. Gottschall, M. H. Grondin, J. Hahn, M. Haupt, G. Heinzelmann, G. Henri, G. Hermann, J. A. Hinton, W. Hofmann, C. Hoischen, T. L. Holch, M. Holler, D. Horns, D. Huber, H. Iwasaki, M. Jamrozy, D. Jankowsky, F. Jankowsky, A. Jardin-Blicq, I. Jung-Richardt, M. A. Kastendieck, K. Katarzyński, M. Katsuragawa, U. Katz, D. Khangulyan, B. Khelifi, J. King, S. Klepser, W. Kluzniak, N. Komin, K. Kosack, D. Kostunin, M. Kreter, G. Lamanna, A. Lemiere, M. Lemoine-Goumard, J. P. Lenain, E. Leser, C. Levy, T. Lohse, I. Lypova, J. Mackey, J. Majumdar, D. Malyshev, V. Marandon, A. Marcowith, A. Mares, C. Mariaud, G. Devesa, R. Marx, G. Maurin, P. J. Meintjes, A. M. W. Mitchell, R. Moderski, M. Mohamed, L. Mohrmann, C. Moore, E. Moulin, J. Muller, T. Murach, S. Nakashima, M. de Naurois, H. Ndiyavala, F. Niederwanger, J. Niemiec, L. Oakes, P. O'Brien, H. Odaka, S. Ohm, E. de Ona Wilhelmi, M. Ostrowski, I. Oya, M. Panter, R. D. Parsons, C. Perennes, P. O. Petrucci, B. Peyaud, Q. Piel, S. Pita, V. Poireau, A. Priyana Noel, D. A. Prokhorov, H. Prokoph, G. Pühlhofer, M. Punch, A. Quirrenbach, S. Raab, R. Rauth, A. Reimer, O. Reimer, Q. Remy, M. Renaud, F. Rieger, L. Rinchiuso, C. Romoli, G. Rowell, B. Rudak, E. Ruiz-Velasco, V. Sahakian, S. Sailer, S. Saito, D. A. Sanchez, A. Santangelo, M. Sasaki, R. Schlickeiser, F. Schüssler, A. Schulz, H. M. Schutte, U. Schwanke, S. Schwemmer, M. Seglar-Arroyo, M. Senniappan, A. S. Seyffert, N. Shafi, K. Shiningayamwe, R. Simoni, A. Sinha, H. Sol, A. Specovius, M. Spir-Jacob, Ł. Stawarz, R. Steenkamp, C. Stegmann, C. Steppa, T. Takahashi, T.

Tavernier, A. M. Taylor, R. Terrier, D. Tiziani, M. Tluczykont, C. Trichard, M. Tsirou, N. Tsuji, R. Tuffs, Y. Uchiyama, D. J. van der Walt, C. van Eldik, C. van Rensburg, B. van Soelen, G. Vasileiadis, J. Veh, C. Venter, P. Vincent, J. Vink, H. J. Völk, T. Vuillaume, Z. Wadiasingh, S. J. Wagner, R. White, A. Wierzcholska, R. Yang, H. Yoneda, M. Zacharias, R. Zanin, A. A. Zdziarski, A. Zech, A. Ziegler, J. Zorn, N. Żywucka, F. de Palma, M. Axelsson, O. J. Roberts, A very-high-energy component deep in the γ-ray burst afterglow. **575** 464–467 (2019).

4. H. E. S. S. Collaboration, H. Abdalla, F. Aharonian, F. Ait Benkhali, E. O. Angüner, C. Arcaro, C. Armand, T. Armstrong, H. Ashkar, M. Backes, V. Baghmanyan, V. Barbosa Martins, A. Barnacka, M. Barnard, Y. Becherini, D. Berge, K. Bernlöhr, B. Bi, E. Bissaldi, M. Böttcher, C. Boisson, J. Bolmont, M. de Bony de Lavergne, M. Breuhaus, F. Brun, P. Brun, M. Bryan, M. Büchele, T. Bulik, T. Bylund, S. Caroff, A. Carosi, S. Casanova, T. Chand, S. Chandra, A. Chen, G. Cotter, M. Curyło, J. Damascene Mbarubucyeye, I. D. Davids, J. Davies, C. Deil, J. Devin, L. Dirson, A. Djannati-Atai, A. Dmytriiev, A. Donath, V. Doroshenko, L. Dreyer, C. Duffy, J. Dyks, K. Egberts, F. Eichhorn, S. Einecke, G. Emery, J. P. Ernenwein, K. Feijen, S. Fegan, A. Fiasson, G. Fichet de Clairfontaine, G. Fontaine, S. Funk, M. Füßling, S. Gabici, Y. A. Gallant, G. Giavitto, L. Giunti, D. Glawion, J. F. Glicenstein, M. H. Grondin, J. Hahn, M. Haupt, G. Hermann, J. A. Hinton, W. Hofmann, C. Hoischen, T. L. Holch, M. Holler, M. Horbe, D. Horns, D. Huber, M. Jamrozy, D. Jankowsky, F. Jankowsky, A. Jardin-Blicq, V. Joshi, I. Jung-Richardt, E. Kasai, M. A. Kastendieck, K. Katarzynski, U. Katz, D. Khangulyan, B. Khelifi, S. Klepser, W. Kluzniak, N. Komin, R. Konno, K. Kosack, D. Kostunin, M. Kreter, G. Lamanna, A. Lemiere, M. Lemoine-Goumard, J. P. Lenain, F. Leuschner, C. Levy, T. Lohse, I. Lypova, J. Mackey, J. Majumdar, D. Malyshev, D. Malyshev, V. Marandon, P. Marchegiani, A. Marcowith, A. Mares, G. Devesa, R. Marx, G. Maurin, P. J. Meintjes, M. Meyer, A. Mitchell, R. Moderski, L. Mohrmann, A. Montanari, C. Moore, P. Morris, E. Moulin, J. Muller, T. Murach, K. Nakashima, A. Nayerhoda, M. de Naurois, H. Ndiyavala, J. Niemiec, L. Oakes, P. O'Brien, H. Odaka, S. Ohm, L. Olivera-Nieto, E. de Ona Wilhelmi, M. Ostrowski, S. Panny, M. Panter, R. D. Parsons, G. Peron, B. Peyaud, Q. Piel, S. Pita, V. Poireau, A. Priyana Noel, D. A. Prokhorov, H. Prokoph, G. Pühlhofer, M. Punch, A. Quirrenbach, S. Raab, R. Rauth, P. Reichherzer, A. Reimer, O. Reimer, Q. Remy, M. Renaud, F. Rieger, L. Rinchiuso, C. Romoli, G. Rowell, B. Rudak, E. Ruiz-Velasco, V. Sahakian, S. Sailer, H. Salzmann, D. A. Sanchez, A. Santangelo, M. Sasaki, M. Scalici, J. Schäfer, F. Schüssler, H. M. Schutte, U. Schwanke, M. Seglar-Arroyo, M. Senniappan, A. S. Seyffert, N. Shafi, J. N. S. Shapopi, K. Shiningayamwe, R. Simoni, A. Sinha, H. Sol, A. Specovius, S. Spencer, M.

Spir-Jacob, L. Stawarz, L. Sun, R. Steenkamp, C. Stegmann, S. Steinmassl, C. Steppa, T. Takahashi, T. Tam, T. Tavernier, A. M. Taylor, R. Terrier, J. H. E. Thiersen, D. Tiziani, M. Tluczykont, L. Tomankova, M. Tsirou, R. Tuffs, Y. Uchiyama, D. J. van der Walt, C. van Eldik, C. van Rensburg, B. van Soelen, G. Vasileiadis, J. Veh, C. Venter, P. Vincent, J. Vink, H. J. Völk, Z. Wadiasingh, S. J. Wagner, J. Watson, F. Werner, R. White, A. Wierzcholska, Yu Wun Wong, A. Yusafzai, M. Zacharias, R. Zanin, D. Zargaryan, A. A. Zdziarski, A. Zech, S. J. Zhu, J. Zorn, S. Zouari, N. Żywucka, P. Evans, K. Page, Revealing x-ray and gamma ray temporal and spectral similarities in the GRB 190829A afterglow. *Science* **372**, 1081–1085 (2021).

5. E. Derishev, T. Piran, The physical conditions of the afterglow implied by MAGIC's sub-TeV observations of GRB 190114C. *Astrophys. J. Lett.* **880**, L27 (2019).
6. X.-Y. Wang, R.-Y. Liu, H.-M. Zhang, S.-Q. Xi, B. Zhang, Synchrotron self-Compton emission from external shocks as the origin of the sub-TeV emission in GRB 180720B and GRB 190114C. *Astrophys. J.* **884**, 117 (2019).
7. S. Lesage, P. Veres, M. S. Briggs, A. Goldstein, D. Kocevski, E. Burns, C. A. Wilson-Hodge, P. N. Bhat, D. Huppenkothen, C. L. Fryer, R. Hamburg, J. Racusin, E. Bissaldi, W. H. Cleveland, S. Dalessi, C. Fletcher, M. M. Giles, B. A. Hristov, C. M. Hui, B. Mailyan, S. Poolakkil, O. J. Roberts, A. von Kienlin, J. Wood, M. Ajello, M. Arimoto, L. Baldini, J. Ballet, M. G. Baring, D. Bastieri, J. Becerra Gonzalez, R. Bellazzini, E. Bissaldi, R. D. Blandford, R. Bonino, P. Bruel, S. Buson, R. A. Cameron, R. Caputo, P. A. Caraveo, E. Cavazzuti, G. Chiaro, N. Cibrario, S. Ciprini, P. Cristarella Orestano, M. Crnogorcevic, A. Cuoco, S. Cutini, F. DAmmando, S. De Gaetano, N. Di Lalla, L. Di Venere, A. Dominguez, S. J. Fegan, E. C. Ferrara, H. Fleischhack, Y. Fukazawa, S. Funk, P. Fusco, G. Galanti, V. Gammaldi, F. Gargano, C. Gasbarra, D. Gasparrini, S. Germani, F. Giacchino, N. Giglietto, R. Gill, M. Giroletti, J. Granot, D. Green, I. A. Grenier, S. Guiriec, M. Gustafsson, E. Hays, J. W. Hewitt, D. Horan, X. Hou, M. Kuss, L. Latronico, A. Laviron, M. Lemoine-Goumard, J. Li, I. Liodakis, F. Longo, F. Loparco, L. Lorusso, M. N. Lovellette, P. Lubrano, S. Maldera, A. Manfreda, G. Marti-Devesa, M. N. Mazziotta, J. E. McEnery, I. Mereu, M. Meyer, P. F. Michelson, T. Mizuno, M. E. Monzani, A. Morselli, I. V. Moskalenko, M. Negro, E. Nuss, N. Omodei, E. Orlando, J. F. Ormes, D. Paneque, G. Panzarini, M. Persic, M. Pesce-Rollins, R. Pillera, F. Piron, H. Poon, T. A. Porter, G. Principe, S. Raino, R. Rando, B. Rani, M. Razzano, S. Razzaque, A. Reimer, O. Reimer, F. Ryde, M. Sanchez-Conde, P. M. Saz Parkinson, L. Scotton, D. Serini, C. Sgro, V. Sharma, E. J. Siskind, G. Spandre, P. Spinelli, H. Tajima, D. F. Torres, J. Valverde, T. Venters, Z. Wadiasingh, K.

Wood, G. Zaharijas, Fermi-GBM Discovery of GRB 221009A: An Extraordinarily Bright GRB from Onset to Afterglow. arXiv e-prints, page arXiv:2303.14172, March 2023.

8. J. A. Kennea, M. Williams, Swift Team, GRB 221009A: Swift detected transient may be GRB. *GRB Coord. Netw.* **32635**, 1 (2022).
9. R. Pillera, E. Bissaldi, N. Omodei, G. La Mura, F. Longo, Fermi-LAT team, GRB 221009A: Fermi-LAT refined analysis. *GRB Coord. Netw.* **32658**, 1 (2022).
10. Z.-H. An, S. Antier, X.-Z. Bi, Q.-C. Bu, C. Cai, X.-L. Cao, A.-E. Camisasca, Z. Chang, G. Chen, L. Chen, T.-X. Chen, W. Chen, Y.-B. Chen, Y. Chen, Y.-P. Chen, M. W. Coughlin, W.-W. Cui, Z.-G. Dai, T. Hussenot-Desenonges, Y.-Q. Du, Y.-Y. Du, Y.-F. Du, C.-C. Fan, F. Frontera, H. Gao, M. Gao, M.-Y. Ge, K. Gong, Y.-D. Gu, J. Guan, D.-Y. Guo, Z.-W. Guo, C. Guidorzi, D.-W. Han, J.-J. He, J.-W. He, D.-J. Hou, Y. Huang, J. Huo, Z. Ji, S.-M. Jia, W.-C. Jiang, D. A. Kann, A. Klotz, L.-D. Kong, L. Lan, A. Li, B. Li, C.-Y. Li, C.-K. Li, G. Li, M.-S. Li, T.-P. Li, W. Li, X.-B. Li, X.-Q. Li, X.-F. Li, Y.-G. Li, Z.-W. Li, J. Liang, X.-H. Liang, J.-Y. Liao, L. Lin, C.-Z. Liu, H.-X. Liu, H.-W. Liu, J.-C. Liu, X.-J. Liu, Y.-Q. Liu, Y.-R. Liu, F.-J. Lu, H. Lu, X.-F. Lu, Q. Luo, T. Luo, B.-Y. Ma, F.-L. Ma, R.-C. Ma, X. Ma, R. Maccary, J.-R. Mao, B. Meng, J.-Y. Nie, M. Orlandini, G. Ou, J.-Q. Peng, W.-X. Peng, R. Qiao, J.-L. Qu, X.-Q. Ren, J.-Y. Shi, Q. Shi, L.-M. Song, X.-Y. Song, J. Su, G.-X. Sun, L. Sun, X.-L. Sun, W.-J. Tan, Y. Tan, L. Tao, Y.-L. Tuo, D. Turpin, J.-Z. Wang, C. Wang, C.-W. Wang, H.-J. Wang, H. Wang, J. Wang, L.-J. Wang, P.-J. Wang, P. Wang, W.-S. Wang, X.-Y. Wang, X.-L. Wang, Y.-S. Wang, Y. Wang, X.-Y. Wen, B.-B. Wu, B.-Y. Wu, H. Wu, S.-H. Xiao, S. Xiao, Y.-X. Xiao, S.-L. Xie, S.-L. Xiong, S.-L. Xiong, D. Xu, H. Xu, Y.-J. Xu, Y.-B. Xu, Y.-C. Xu, Y.-P. Xu, W.-C. Xue, S. Yang, Y.-J. Yang, Z.-X. Yang, W.-T. Ye, Q.-B. Yi, S.-X. Yi, Q.-Q. Yin, Y. You, Y.-W. Yu, W. Yu, W.-H. Yu, M. Zeng, B. Zhang, B.-B. Zhang, D.-L. Zhang, F. Zhang, H.-M. Zhang, J. Zhang, L. Zhang, P. Zhang, P. Zhang, S. Zhang, S.-N. Zhang, W.-C. Zhang, X.-F. Zhang, X.-L. Zhang, Y.-Q. Zhang, Y.-T. Zhang, Y.-F. Zhang, Y.-H. Zhang, Z. Zhang, G.-Y. Zhao, H.-S. Zhao, H.-Y. Zhao, Q.-X. Zhao, S.-J. Zhao, X.-Y. Zhao, X.-F. Zhao, Y. Zhao, C. Zheng, S.-J. Zheng, D.-K. Zhou, X. Zhou, X.-C. Zhu, Insight-HXMT and GECAM-C observations of the brightest-of-all-time GRB 221009A. *arXiv e-prints*, page arXiv:2303.01203, March 2023.
11. D. Frederiks, D. Svinkin, A. L. Lysenko, S. Molkov, A. Tsvetkova, M. Ulanov, A. Ridnaia, A. A. Lutovinov, I. Lapshov, A. Tkachenko, V. Levin, Properties of the extremely energetic GRB221009A from Konus-\WIND/ and \SRG/ART-XC observations. arXiv e-prints, page arXiv:2302.13383, February 2023.

12. A. de Ugarte Postigo, L. Izzo, G. Pugliese, D. Xu, B. Schneider, J. P. U. Fynbo, N. R. Tanvir, D. B. Malesani, A. Saccardi, D. A. Kann, K. Wiersema, B. P. Gompertz, C. C. Thoene, A. J. Levan, Stargate Collaboration, GRB 221009A: Redshift from X-shooter/VLT. *GRB Coord. Netw.* **32648**, 1 (2022).
13. X.-H. Ma, Y.-J. Bi, Z. Cao, M.-J. Chen, S.-Z. Chen, Y.-D. Cheng, G.-H. Gong, Min-Hao Gu, H.-H. He, C. Hou, W.-H. Huang, X.-T. Huang, C. Liu, O. Shchegolev, X.-D. Sheng, Y. Stenkin, C.-Y. Wu, H.-R. Wu, S. Wu, G. Xiao, Z.-G. Yao, S.-S. Zhang, Y. Zhang, X. Zuo, Chapter 1 LHAASO Instruments and Detector technology. *Chin. Phys. C* **46**, 030001 (2022).
14. Y. Huang, S. Hu, S. Chen, M. Zha, C. Liu, Z. Yao, Zhen Cao, The Lhaaso Experiment, LHAASO observed GRB 221009A with more than 5000 VHE photons up to around 18 TeV *GRB Coord. Netw.* **32677**, 1, (2022).
15. LHAASO Collaboration, Z. Cao, F. Aharonian, Q. An, A. Axikegu, L. X. Bai, Y. X. Bai, Y. W. Bao, D. Bastieri, X. J. Bi, Y. J. Bi, J. T. Cai, Q. Cao, W. Y. Cao, Z. Cao, J. Chang, J. F. Chang, E. S. Chen, L. Chen, L. Chen, L. Chen, M. J. Chen, M. L. Chen, Q. H. Chen, S. H. Chen, S. Z. Chen, T. L. Chen, Y. Chen, H. L. Cheng, N. Cheng, Y. D. Cheng, S. W. Cui, X. H. Cui, Y. D. Cui, B. Z. Dai, H. L. Dai, D. Danzengluobu, D. Della Volpe, X. Q. Dong, K. K. Duan, J. H. Fan, Y. Z. Fan, J. Fang, K. Fang, C. F. Feng, L. Feng, S. H. Feng, X. T. Feng, Y. L. Feng, B. Gao, C. D. Gao, L. Q. Gao, Q. Gao, W. Gao, W. K. Gao, M. M. Ge, L. S. Geng, G. H. Gong, Q. B. Gou, M. H. Gu, F. L. Guo, X. L. Guo, Y. Q. Guo, Y. Y. Guo, Y. A. Han, H. H. He, H. N. He, J. Y. He, X. B. He, Y. He, M. Heller, Y. K. Hor, B. W. Hou, C. Hou, X. Hou, H. B. Hu, Q. Hu, S. C. Hu, D. H. Huang, T. Q. Huang, W. J. Huang, X. T. Huang, Z. C. Huang, X. L. Ji, H. Y. Jia, K. Jia, K. Jiang, X. W. Jiang, Z. J. Jiang, M. Jin, M. M. Kang, T. Ke, D. Kuleshov, K. Kurinov, B. B. Li, C. Li, C. Li, D. Li, F. Li, H. B. Li, H. C. Li, H. Y. Li, J. Li, J. Li, J. Li, K. Li, W. L. Li, W. L. Li, X. R. Li, X. Li, Y. Z. Li, Z. Li, Z. Li, E. W. Liang, Y. F. Liang, S. J. Lin, B. Liu, C. Liu, D. Liu, H. Liu, H. D. Liu, J. Liu, J. L. Liu, J. L. Liu, J. S. Liu, J. Y. Liu, M. Y. Liu, R. Y. Liu, S. M. Liu, W. Liu, Y. Liu, Y. N. Liu, W. J. Long, R. Lu, Q. Luo, H. K. Lv, B. Q. Ma, L. L. Ma, X. H. Ma, J. R. Mao, Z. Min, W. Mitthumsiri, Y. C. Nan, Z. W. Ou, B. Y. Pang, P. Pattarakijwanich, Z. Y. Pei, M. Y. Qi, Y. Q. Qi, B. Q. Qiao, J. J. Qin, D. Ruffolo, A. Saiz, C. Y. Shao, L. Shao, O. Shchegolev, X. D. Sheng, H. C. Song, Y. V. Stenkin, V. Stepanov, Y. Su, Q. N. Sun, X. N. Sun, Z. B. Sun, P. H. T. Tam, Z. B. Tang, W. W. Tian, C. Wang, C. B. Wang, G. W. Wang, H. G. Wang, H. H. Wang, J. C. Wang, J. S. Wang, K. Wang, L. P. Wang, L. Y. Wang, P. H. Wang, R. Wang, W. Wang, X. G. Wang, Y. D. Wang, Y. J. Wang, Z. H. Wang, Z. X. Wang, Z. Wang, D. M. Wei, J. J. Wei, Y. J. Wei, T. Wen, C. Y. Wu, H. R. Wu, S. Wu, X. F. Wu, Y. S. Wu, S. Q. Xi, J. Xia, J. J. Xia, G. M. Xiang, D. X. Xiao,

G. Xiao, G. G. Xin, Y. L. Xin, Y. Xing, Z. Xiong, D. L. Xu, R. F. Xu, R. X. Xu, L. Xue, D. H. Yan, J. Z. Yan, T. Yan, C. W. Yang, F. Yang, F. F. Yang, H. W. Yang, J. Y. Yang, L. L. Yang, M. J. Yang, R. Z. Yang, S. B. Yang, Y. H. Yao, Y. M. Ye, L. Q. Yin, N. Yin, X. H. You, Z. Y. You, Y. H. Yu, Q. Yuan, H. Yue, H. D. Zeng, T. X. Zeng, W. Zeng, Z. K. Zeng, B. Zhang, B. B. Zhang, F. Zhang, H. M. Zhang, H. Y. Zhang, J. L. Zhang, L. X. Zhang, L. Zhang, P. F. Zhang, P. P. Zhang, R. Zhang, S. B. Zhang, S. R. Zhang, S. S. Zhang, X. Zhang, X. P. Zhang, Y. F. Zhang, Y. Zhang, Y. Zhang, B. Zhao, J. Zhao, L. Zhao, L. Z. Zhao, S. P. Zhao, F. Zheng, B. Zhou, H. Zhou, J. N. Zhou, P. Zhou, R. Zhou, X. X. Zhou, C. G. Zhu, F. R. Zhu, H. Zhu, K. J. Zhu, X. Zuo, A tera-electron volt afterglow from a narrow jet in an extremely bright gamma-ray burst. *Science* **380**, 1390–1396 (2023).

16. F. Aharonian, Q. An, Axikegu, L. X. Bai, Y. X. Bai, Y. W. Bao, D. Bastieri, X. J. Bi, Y. J. Bi, H. Cai, J. T. Cai, Z. Cao, Z. Cao, J. Chang, J. F. Chang, X. C. Chang, B. M. Chen, J. Chen, L. Chen, L. Chen, L. Chen, M. J. Chen, M. L. Chen, Q. H. Chen, S. H. Chen, S. Z. Chen, T. L. Chen, X. L. Chen, Y. Chen, N. Cheng, Y. D. Cheng, S. W. Cui, X. H. Cui, Y. D. Cui, B. Z. Dai, H. L. Dai, Z. G. Dai, Danzengluobu, D. Della Volpe, B. D'ettorre Piazzoli, X. J. Dong, J. H. Fan, Y. Z. Fan, Z. X. Fan, J. Fang, K. Fang, C. F. Feng, L. Feng, S. H. Feng, Y. L. Feng, B. Gao, C. D. Gao, Q. Gao, W. Gao, M. M. Ge, L. S. Geng, G. H. Gong, Q. B. Gou, M. H. Gu, J. G. Guo, X. L. Guo, Y. Q. Guo, Y. Y. Guo, Y. A. Han, H. H. He, H. N. He, J. C. He, S. L. He, X. B. He, Y. He, M. Heller, Y. K. Hor, C. Hou, X. Hou, H. B. Hu, S. Hu, S. C. Hu, X. J. Hu, D. H. Huang, Q. L. Huang, W. H. Huang, X. T. Huang, Z. C. Huang, F. Ji, X. L. Ji, H. Y. Jia, K. Jiang, Z. J. Jiang, C. Jin, D. Kuleshov, K. Levochkin, B. B. Li, C. Li, C. Li, F. Li, H. B. Li, H. C. Li, H. Y. Li, J. Li, K. Li, W. L. Li, X. Li, X. Li, X. R. Li, Y. Li, Y. Z. Li, Z. Li, Z. Li, E. W. Liang, Y. F. Liang, S. J. Lin, B. Liu, C. Liu, D. Liu, H. Liu, H. D. Liu, J. Liu, J. L. Liu, J. S. Liu, J. Y. Liu, M. Y. Liu, R. Y. Liu, S. M. Liu, W. Liu, Y. N. Liu, Z. X. Liu, W. J. Long, R. Lu, H. K. Lv, B. Q. Ma, L. L. Ma, X. H. Ma, J. R. Mao, A. Masood, W. Mitthumsiri, T. Montaruli, Y. C. Nan, B. Y. Pang, P. Pattarakijwanich, Z. Y. Pei, M. Y. Qi, D. Ruffolo, V. Rulev, A. Sáiz, L. Shao, O. Shchegolev, X. D. Sheng, J. R. Shi, H. C. Song, Y. V. Stenkin, V. Stepanov, Q. N. Sun, X. N. Sun, Z. B. Sun, P. H. T. Tam, Z. B. Tang, W. W. Tian, B. D. Wang, C. Wang, H. Wang, H. G. Wang, J. C. Wang, J. S. Wang, L. P. Wang, L. Y. Wang, R. N. Wang, W. Wang, W. Wang, X. G. Wang, X. J. Wang, X. Y. Wang, Y. D. Wang, Y. J. Wang, Y. P. Wang, Z. Wang, Z. Wang, Z. H. Wang, Z. X. Wang, D. M. Wei, J. J. Wei, Y. J. Wei, T. Wen, C. Y. Wu, H. R. Wu, S. Wu, W. X. Wu, X. F. Wu, S. Q. Xi, J. Xia, J. J. Xia, G. M. Xiang, G. Xiao, H. B. Xiao, G. G. Xin, Y. L. Xin, Y. Xing, D. L. Xu, R. X. Xu, L. Xue, D. H. Yan, C. W. Yang, F. F. Yang, J. Y. Yang, L. L. Yang, M. J. Yang, R. Z. Yang, S.

B. Yang, Y. H. Yao, Z. G. Yao, Y. M. Ye, L. Q. Yin, N. Yin, X. H. You, Z. Y. You, Y. H. Yu, Q. Yuan, H. D. Zeng, T. X. Zeng, W. Zeng, Z. K. Zeng, M. Zha, X. X. Zhai, B. B. Zhang, H. M. Zhang, H. Y. Zhang, J. L. Zhang, J. W. Zhang, L. Zhang, L. Zhang, L. X. Zhang, P. F. Zhang, P. P. Zhang, R. Zhang, S. R. Zhang, S. S. Zhang, X. Zhang, X. P. Zhang, Y. Zhang, Y. Zhang, Y. F. Zhang, Y. L. Zhang, B. Zhao, J. Zhao, L. Zhao, L. Z. Zhao, S. P. Zhao, F. Zheng, Y. Zheng, B. Zhou, H. Zhou, J. N. Zhou, P. Zhou, R. Zhou, X. X. Zhou, C. G. Zhu, F. R. Zhu, H. Zhu, K. J. Zhu, X. Zuo; Lhaaso Collaboration, Observation of the Crab Nebula with LHAASO-KM2A—A performance study. *Chin. Phys. C* **45**, 025002 (2021).

17. A. Saldana-Lopez, A. Dominguez, P. G. Pérez-González, J. Finke, M. Ajello, J. R. Primack, V. S. Paliya, A. Desai, An observational determination of the evolving extragalactic background light from the multiwavelength HST/CANDELS survey in the Fermi and CTA era. *Mont. Not. R. Astron. Soc.* **507**, 5144–5160 (2021).
18. R. C. Gilmore, R. S. Somerville, J. R. Primack, A. Dominguez, Semi-analytic modelling of the extragalactic background light and consequences for extragalactic gamma-ray spectra. *Mont. Not. R. Astron. Soc.* **422**, 3189–3207 (2012).
19. A. Dominguez, J. R. Primack, D. J. Rosario, F. Prada, R. C. Gilmore, S. M. Faber, D. C. Koo, R. S. Somerville, M. A. Pérez-Torres, P. Pérez-González, J. S. Huang, M. Davis, P. Guhathakurta, P. Barmby, C. J. Conselice, M. Lozano, J. A. Newman, M. C. Cooper, Extragalactic background light inferred from AEGIS galaxy-SED-type fractions. *Mon. Not. R. Astron. Soc. Lett.* **410**, 2556–2578 (2011).
20. J. D. Finke, S. Razzaque, C. D. Dermer, Modeling the Extragalactic Background Light from Stars and Dust. *Astrophys. J.* **712**, 238–249 (2010).
21. F. A. Aharonian, A. G. Akhperjanian, J. A. Barrio, K. Bernlöhr, H. Bojahr, I. Calle, J. L. Contreras, J. Cortina, A. Daum, T. Deckers, S. Denninghoff, V. Fonseca, J. C. Gonzalez, G. Heinzelmann, M. Hemberger, G. Hermann, M. Heß, A. Heusler, W. Hofmann, H. Hohl, D. Horns, A. Ibarra, R. Kankanyan, J. Kettler, C. Köhler, A. Konopelko, H. Kornmeyer, M. Kestel, D. Kranich, H. Krawczynski, H. Lampeitl, A. Lindner, E. Lorenz, N. Magnussen, H. Meyer, R. Mirzoyan, A. Moralejo, L. Padilla, M. Panter, D. Petry, R. Plaga, A. Plyasheshnikov, J. Prahl, G. Pühlhofer, G. Rauterberg, C. Renault, W. Rhode, A. Röhring, V. Sahakian, M. Samorski, D. Schmele, F. Schröder, W. Stamm, H. J. Völk, B. Wiebel-Sooth, C. Wiedner, M. Willmer, W. Wittek, The time averaged TeV

energy spectrum of MKN 501 of the extraordinary 1997 outburst as measured with the stereoscopic Cherenkov telescope system of HEGRA. *Astron Astrophys* **349**, 11–28 (1999).

22. F. A. Aharonian, A. N. Timokhin, A. V. Plyasheshnikov, On the origin of highest energy gamma-rays from Mkn 501. *Astron Astrophys* **384**, 834–847 (2002).
23. F. A. Aharonian, TeV blazars and cosmic infrared background radiation. In 27th International Cosmic Ray Conference (ICRC27), volume 27 of International Cosmic Ray Conference, page 250, January 2001.
24. S. Berta, B. Magnelli, D. Lutz, B. Altieri, H. Aussel, P. Andreani, O. Bauer, A. Bongiovanni, A. Cava, J. Cepa, A. Cimatti, E. Daddi, H. Dominguez, D. Elbaz, H. Feuchtgruber, N. M. Förster Schreiber, R. Genzel, C. Gruppioni, R. Katterloher, G. Magdis, R. Maiolino, R. Nordon, A. M. Pérez García, A. Poglitsch, P. Popesso, F. Pozzi, L. Riguccini, G. Rodighiero, A. Saintonge, P. Santini, M. Sanchez-Portal, L. Shao, E. Sturm, L. J. Tacconi, I. Valtchanov, M. Wetzstein, E. Wieprecht, Dissecting the cosmic infra-red background withHerschel/PEP. *Astron. Astrophys.* **518**, L30 (2010).
25. V. Anastassopoulos, New CAST Limit on the Axion-Photon Interaction. *Nature Phys.* **13**, 584–590 (2017).
26. A. Abramowski, F. Acero, F. Aharonian, F. Ait Benkhali, A. G. Akhperjanian, E. Angüner, G. Anton, S. Balenderan, A. Balzer, A. Barnacka, Y. Becherini, J. Becker Tjus, K. Bernlöhr, E. Birsin, E. Bissaldi, J. Biteau, C. Boisson, J. Bolmont, P. Bordas, J. Brucker, F. Brun, P. Brun, T. Bulik, S. Carrigan, S. Casanova, M. Cerruti, P. M. Chadwick, R. Chalme-Calvet, R. C. G. Chaves, A. Cheesebrough, M. Chrétien, S. Colafrancesco, G. Cologna, J. Conrad, C. Couturier, M. Dalton, M. K. Daniel, I. D. Davids, B. Degrange, C. Deil, P. deWilt, H. J. Dickinson, A. Djannati-Ataï, W. Domainko, L. O.'C. Drury, G. Dubus, K. Dutson, J. Dyks, M. Dyrda, T. Edwards, K. Egberts, P. Eger, P. Espigat, C. Farnier, S. Fegan, F. Feinstein, M. V. Fernandes, D. Fernandez, A. Fiasson, G. Fontaine, A. Förster, M. Füßling, M. Gajdus, Y. A. Gallant, T. Garrigoux, H. Gast, B. Giebels, J. F. Glicenstein, D. Göring, M. H. Grondin, M. Grudzińska, S. Häffner, J. D. Hague, J. Hahn, J. Harris, G. Heinzelmann, G. Henri, G. Hermann, O. Hervet, A. Hillert, J. A. Hinton, W. Hofmann, P. Hofverberg, M. Holler, D. Horns, A. Jacholkowska, C. Jahn, M. Jamrozy, M. Janiak, F. Jankowsky, I. Jung, M. A. Kastendieck, K. Katarzyński, U. Katz, S. Kaufmann, B. Khélifi, M. Kieffer, S. Klepser, D. Klochkov, W. Kluźniak, T. Kneiske, D. Kolitzus, N. Komin, K. Kosack, S. Krakau, F. Krayzel, P. P. Krüger, H. Laffon, G. Lamanna, J. Lefaucheur, M. Lemoine-Goumard, J. P. Lenain, D. Lennarz, T. Lohse, A. Lopatin, C. C. Lu, V. Marandon, A. Marcowith, R. Marx, G. Maurin, N. Maxted, M. Mayer, T. J. L.

McComb, M. C. Medina, J. Méhault, U. Menzler, M. Meyer, R. Moderski, M. Mohamed, E. Moulin, T. Murach, C. L. Naumann, M. de Naurois, D. Nedbal, J. Niemiec, S. J. Nolan, L. Oakes, S. Ohm, E. de Oña Wilhelmi, B. Opitz, M. Ostrowski, I. Oya, M. Panter, R. D. Parsons, M. Paz Arribas, N. W. Pekeur, G. Pelletier, J. Perez, P. O. Petrucci, B. Peyaud, S. Pita, H. Poon, G. Pühlhofer, M. Punch, A. Quirrenbach, S. Raab, M. Raue, A. Reimer, O. Reimer, M. Renaud, R. de los Reyes, F. Rieger, L. Rob, S. Rosier-Lees, G. Rowell, B. Rudak, C. B. Rulten, V. Sahakian, D. A. Sanchez, A. Santangelo, R. Schlickeiser, F. Schüssler, A. Schulz, U. Schwanke, S. Schwarzburg, S. Schwemmer, H. Sol, G. Spengler, F. Spieß, L. Stawarz, R. Steenkamp, C. Stegmann, F. Stinzing, K. Stycz, I. Sushch, A. Szostek, J. P. Tavernet, R. Terrier, M. Tluczykont, C. Trichard, K. Valerius, C. van Eldik, G. Vasileiadis, C. Venter, A. Viana, P. Vincent, H. J. Völk, F. Volpe, M. Vorster, S. J. Wagner, P. Wagner, M. Ward, M. Weidinger, Q. Weitzel, R. White, A. Wierzcholska, P. Willmann, A. Wörnlein, D. Wouters, M. Zacharias, A. Zajczyk, A. A. Zdziarski, A. Zech, H. S. Zechlin, Constraints on axionlike particles with H.E.S.S. from the irregularity of the PKS2155−304 energy spectrum. *Phys. Rev. D* **88**, 102003 (2013).

27. H.-J. Li, J.-G. Guo, X.-J. Bi, S.-J. Lin, P.-F. Yin, Limits on axion-like particles from Mrk 421 with 4.5-year period observations by ARGO-YBJ and Fermi-LAT. *Phys. Rev. D* **103**, 083003 (2021).
28. Z. Cao, F. Aharonian, Q. An, Axikegu, L. X. Bai, Y. X. Bai, Y. W. Bao, D. Bastieri, X. J. Bi, Y. J. Bi, H. Cai, J. T. Cai, Zhe Cao, J. Chang, J. F. Chang, B. M. Chen, E. S. Chen, J. Chen, Liang Chen, Liang Chen, Long Chen, M. J. Chen, M. L. Chen, Q. H. Chen, S. H. Chen, S. Z. Chen, T. L. Chen, X. L. Chen, Y. Chen, N. Cheng, Y. D. Cheng, S. W. Cui, X. H. Cui, Y. D. Cui, B. D'Ettorre Piazzoli, B. Z. Dai, H. L. Dai, Z. G. Dai, Danzengluobu, D. della Volpe, X. J. Dong, K. K. Duan, J. H. Fan, Y. Z. Fan, Z. X. Fan, J. Fang, K. Fang, C. F. Feng, L. Feng, S. H. Feng, Y. L. Feng, B. Gao, C. D. Gao, L. Q. Gao, Q. Gao, W. Gao, M. M. Ge, L. S. Geng, G. H. Gong, Q. B. Gou, M. H. Gu, F. L. Guo, J. G. Guo, X. L. Guo, Y. Q. Guo, Y. Y. Guo, Y. A. Han, H. H. He, H. N. He, J. C. He, S. L. He, X. B. He, Y. He, M. Heller, Y. K. Hor, C. Hou, X. Hou, H. B. Hu, S. Hu, S. C. Hu, X. J. Hu, D. H. Huang, Q. L. Huang, W. H. Huang, X. T. Huang, X. Y. Huang, Z. C. Huang, F. Ji, X. L. Ji, H. Y. Jia, K. Jiang, Z. J. Jiang, C. Jin, T. Ke, D. Kuleshov, K. Levochkin, B. B. Li, Cheng Li, Cong Li, F. Li, H. B. Li, H. C. Li, H. Y. Li, Jian Li, Jie Li, K. Li, W. L. Li, X. R. Li, Xin Li, Xin Li, Y. Li, Y. Z. Li, Zhe Li, Zhuo Li, E. W. Liang, Y. F. Liang, S. J. Lin, B. Liu, C. Liu, D. Liu, H. Liu, H. D. Liu, J. Liu, J. L. Liu, J. S. Liu, J. Y. Liu, M. Y. Liu, R. Y. Liu, S. M. Liu, W. Liu, Y. Liu, Y. N. Liu, Z. X. Liu, W. J. Long, R. Lu, H. K. Lv, B. Q. Ma, L. L. Ma, X. H. Ma, J. R. Mao, A. Masood, Z. Min, W. Mitthumsiri, T. Montaruli, Y. C. Nan, B.

Y. Pang, P. Pattarakijwanich, Z. Y. Pei, M. Y. Qi, Y. Q. Qi, B. Q. Qiao, J. J. Qin, D. Ruffolo, V. Rulev, A. Sáiz, L. Shao, O. Shchegolev, X. D. Sheng, J. R. Shi, H. C. Song, Yu. V. Stenkin, V. Stepanov, Y. Su, Q. N. Sun, X. N. Sun, Z. B. Sun, P. H. T. Tam, Z. B. Tang, W. W. Tian, B. D. Wang, C. Wang, H. Wang, H. G. Wang, J. C. Wang, J. S. Wang, L. P. Wang, L. Y. Wang, R. N. Wang, W. Wang, W. Wang, X. G. Wang, X. J. Wang, X. Y. Wang, Y. Wang, Y. D. Wang, Y. J. Wang, Y. P. Wang, Z. H. Wang, Z. X. Wang, Zhen Wang, Zheng Wang, D. M. Wei, J. J. Wei, Y. J. Wei, T. Wen, C. Y. Wu, H. R. Wu, S. Wu, W. X. Wu, X. F. Wu, S. Q. Xi, J. Xia, J. J. Xia, G. M. Xiang, D. X. Xiao, G. Xiao, H. B. Xiao, G. G. Xin, Y. L. Xin, Y. Xing, D. L. Xu, R. X. Xu, L. Xue, D. H. Yan, J. Z. Yan, C. W. Yang, F. F. Yang, J. Y. Yang, L. L. Yang, M. J. Yang, R. Z. Yang, S. B. Yang, Y. H. Yao, Z. G. Yao, Y. M. Ye, L. Q. Yin, N. Yin, X. H. You, Z. Y. You, Y. H. Yu, Q. Yuan, H. D. Zeng, T. X. Zeng, W. Zeng, Z. K. Zeng, M. Zha, X. X. Zhai, B. B. Zhang, H. M. Zhang, H. Y. Zhang, J. L. Zhang, J. W. Zhang, L. X. Zhang, Li Zhang, Lu Zhang, P. F. Zhang, P. P. Zhang, R. Zhang, S. R. Zhang, S. S. Zhang, X. Zhang, X. P. Zhang, Y. F. Zhang, Y. L. Zhang, Yi Zhang, Yong Zhang, B. Zhao, J. Zhao, L. Zhao, L. Z. Zhao, S. P. Zhao, F. Zheng, Y. Zheng, B. Zhou, H. Zhou, J. N. Zhou, P. Zhou, R. Zhou, X. X. Zhou, C. G. Zhu, F. R. Zhu, H. Zhu, K. J. Zhu, X. Zuo; LHAASO Collaboration, Exploring Lorentz invariance violation from ultrahigh-energy γ rays observed by LHAASO. *Phys. Rev. Lett.* **128**, 051102 (2022).

29. M. Ackermann, A limit on the variation of the speed of light arising from quantum gravity effects. *Nature* **462**, 331–334 (2009).
30. Ehud Nakar, Klein–nishina effects on optically thin synchrotron and synchrotron self-compton spectrum. *Astrophys. J.* **703**, 675–691 (2009).
31. B. Theodore Zhang, K. Murase, K. Ioka, D. Song, C. Yuan, P. Mészáros, External inverse-compton and proton synchrotron emission from the reverse shock as the origin of VHE gamma rays from the hyper-bright GRB 221009A. *Astrophys. J. Lett.* **947**, L14 (2023).
32. S. Das, S. Razzaque, Ultrahigh-energy cosmic-ray signature in GRB 221009A. *Astron. Astrophys.* **670**, L12 (2023).
33. D. Khangulyan, A. M. Taylor, F. Aharonian, The formation of hard very high energy spectra from gamma-ray burst afterglows via two-zone synchrotron self-Compton emission. *Astrophys. J.* **947**, 87 (2023).
34. K. Asano, P. Mészáros, Ultrahigh-energy cosmic ray production by turbulence in gamma-ray burst jets and cosmogenic neutrinos. *Phys. Rev. D*, **94**, 023005 (2016).

35. M. G. Hauser, R. G. Arendt, T. Kelsall, E. Dwek, N. Odegard, J. L. Weiland, H. T. Freudenreich, W. T. Reach, R. F. Silverberg, S. H. Moseley, Y. C. Pei, P. Lubin, J. C. Mather, R. A. Shafer, G. F. Smoot, R. Weiss, D. T. Wilkinson, E. L. Wright, The COBE diffuse infrared background experiment search for the cosmic infrared background. I. Limits and detections. *Astrophys. J.* **508**, 25–43 (1998).
36. G. Lagache, L. M. Haffner, R. J. Reynolds, S. L. Tufte, Evidence for dust emission in the warm ionised medium using wham data. *Astron. Astrophys.* **354**, 247 (2000).
37. J. P. Gardner, S. A. Baum, T. M. Brown, C. M. Carollo, J. Christensen, I. Dashevsky, M. E. Dickinson, B. R. Espey, H. C. Ferguson, A. S. Fruchter, A. M. Gonnella, R. A. Gonzalez-Lopezlira, R. N. Hook, M. E. Kaiser, C. L. Martin, K. C. Sahu, S. Savaglio, T. E. Smith, H. I. Teplitz, R. E. Williams, J. Wilson, The hubble deep field south - stis imaging. *Astron. J.* **119**, 486–508 (2000).
38. D. Elbaz, C. Cesarsky, P. Chanial, H. Aussel, A. Franceschini, D. Fadda, R. Chary, The Bulk of the cosmic infrared background resolved by ISOCAM. *Astron. Astrophys.* **384**, 848–865 (2002).
39. G. G. Fazio, M. L. N. Ashby, P. Barmby, J. L. Hora, J. S. Huang, M. A. Pahre, Z. Wang, S. P. Willner, R. G. Arendt, S. H. Moseley, M. Brodwin, P. Eisenhardt, D. Stern, E. V. Tollestrup, E. L. Wright, Number counts at 3 μm $< \lambda <$ 10 μm from the Spitzer Space Telescope. *Astrophys. J. Suppl.* **154**, 39–43 (2004).
40. C. Kevin Xu, Number counts of GALEX sources in far-ultraviolet (1530 Å) and near-ultraviolet (2310 Å) bands. *Astrophys. J. Lett.* **619**, L11–L14 (2005).
41. M. Bethermin, H. Dole, A. Beelen, H. Aussel, Spitzer deep and wide legacy mid- and far-infrared number counts and lower limits of cosmic infrared background. *Astron. Astrophys.* **512**, A78 (2010).
42. S. Matsuura, M. Shirahata, M. Kawada, T. T. Takeuchi, D. Burgarella, D. L. Clements, W. S. Jeong, H. Hanami, S. A. Khan, H. Matsuhara, T. Nakagawa, S. Oyabu, C. P. Pearson, A. Pollo, S. Serjeant, T. Takagi, G. J. White, Detection of the cosmic far-infrared background in AKARI Deep Field South. *Astrophys. J.* **737**, 2 (2011).
43. E. N. Voyer, J. P. Gardner, H. I. Teplitz, B. D. Siana, D. F. de Mello, Far-ultraviolet number counts of field galaxies. *Astrophys. J.* **736**, 80 (2011).
44. M. Zemcov, J. Smidt, T. Arai, J. Bock, A. Cooray, Y. Gong, M. G. Kim, P. Korngut, A. Lam, D. H. Lee, T. Matsumoto, S. Matsuura, U. W. Nam, G. Roudier, K. Tsumura, T. Wada, On the origin of near-infrared extragalactic background light anisotropy. *Science* **346**, 732–735 (2014).

45. S. P. Driver, S. K. Andrews, L. J. Davies, A. S. G. Robotham, A. H. Wright, R. A. Windhorst, S. Cohen, K. Emig, R. A. Jansen, L. Dunne, Measurements of extragalactic background light from the far UV to the far IR from deep ground- and space-based galaxy counts. *Astrophys. J.* **827**, 1 (2016).
46. Douglas P. Finkbeiner, Marc Davis, David J. Schlegel, Detection of a far-infrared excess with dirbe at 60 and 100 microns. *Astrophys. J.* **544**, 81–97 (2000).
47. Piero Madau, Lucia Pozzetti, Deep galaxy counts, extragalactic background light, and the stellar baryon budget. *Mon. Not. Roy. Astron. Soc.* **312**, L9–L15 (2000).
48. L. Metcalfe, J. P. Kneib, B. McBreen, B. Altieri, A. Biviano, M. Delaney, D. Elbaz, M. F. Kessler, K. Leech, K. Okumura, S. Ott, R. Perez-Martinez, C. Sanchez-Fernandez, B. Schulz, An ISOCAM survey through gravitationally lensing galaxy clusters. *Astron. Astrophys.* **407**, 791–822 (2003).
49. Casey Papovich, H. Dole, E. Egami, E. le Floc'h, P. G. Perez-Gonzalez, A. Alonso-Herrero, L. Bai, C. A. Beichman, M. Blaylock, C. W. Engelbracht, K. D. Gordon, D. C. Hines, K. A. Misselt, J. E. Morrison, J. Mould, J. Muzerolle, G. Neugebauer, P. L. Richards, G. H. Rieke, M. J. Rieke, J. R. Rigby, K. Y. L. Su, E. T. Young, The 24 micron source counts in Deep Spitzer Space Telescope Surveys. *Astrophys. J. Suppl.* **154**, 70–74 (2004).
50. D. T. Frayer, M. T. Huynh, R. Chary, M. Dickinson, D. Elbaz, D. Fadda, J. A. Surace, H. I. Teplitz, L. Yan, B. Mobasher, Spitzer 70 micron source counts in GOODS-North. *Astrophys. J. Lett.* **647**, L9–L12 (2006).
51. R. C. Keenan, A. J. Barger, L. L. Cowie, W.-H. Wang, The resolved near-infrared extragalactic background. *Astrophys. J.* **723**, 40–46 (2010).
52. Y. Matsuoka, N. Ienaka, K. Kawara, S. Oyabu, Cosmic optical background: The view from Pioneer 10/11. *Astrophys. J.* **736**, 119 (2011).
53. A. Penin, G. Lagache, A. Noriega-Crepo, J. Grain, M.-A. Miville-Deschenes, N. Ponthieu, P. Martin, K. Blagrave, F. J. Lockman, An accurate measurement of the anisotropies and mean level of the Cosmic Infrared Background at 100 μm and 160 μm. *Astron. Astrophys.*, **543** A123 (2012).
54. K. Mattila, P. Väisänen, K. Lehtinen, G. von Appen-Schnur, C. Leinert, Extragalactic background light: A measurement at 400 nm using dark cloud shadow - II. Spectroscopic separation of the dark cloud's light, and results. *Monthly Notices of the Royal Astronomical Society* **470**, 2152–2169 (2017).
55. F. Aharonian, Q. An, Axikegu, L. X. Bai, Y. X. Bai, Y. W. Bao, D. Bastieri, X. J. Bi, Y. J. Bi, H. Cai, J. T. Cai, Z. Cao, Z. Cao, J. Chang, J. F. Chang, X. C. Chang, B. M. Chen, J. Chen, L. Chen, L. Chen, L. Chen, M. J. Chen, M. L. Chen, Q. H. Chen, S. H. Chen, S. Z. Chen, T. L. Chen, X. L. Chen, Y.

Chen, N. Cheng, Y. D. Cheng, S. W. Cui, X. H. Cui, Y. D. Cui, B. Z. Dai, H. L. Dai, Z. G. Dai, Danzengluobu, D. Della Volpe, B. D'ettorre Piazzoli, X. J. Dong, J. H. Fan, Y. Z. Fan, Z. X. Fan, J. Fang, K. Fang, C. F. Feng, L. Feng, S. H. Feng, Y. L. Feng, B. Gao, C. D. Gao, Q. Gao, W. Gao, M. M. Ge, L. S. Geng, G. H. Gong, Q. B. Gou, M. H. Gu, J. G. Guo, X. L. Guo, Y. Q. Guo, Y. Y. Guo, Y. A. Han, H. H. He, H. N. He, J. C. He, S. L. He, X. B. He, Y. He, M. Heller, Y. K. Hor, C. Hou, X. Hou, H. B. Hu, S. Hu, S. C. Hu, X. J. Hu, D. H. Huang, Q. L. Huang, W. H. Huang, X. T. Huang, Z. C. Huang, F. Ji, X. L. Ji, H. Y. Jia, K. Jiang, Z. J. Jiang, C. Jin, D. Kuleshov, K. Levochkin, B. B. Li, C. Li, C. Li, F. Li, H. B. Li, H. C. Li, H. Y. Li, J. Li, K. Li, W. L. Li, X. Li, X. Li, X. R. Li, Y. Li, Y. Z. Li, Z. Li, Z. Li, E. W. Liang, Y. F. Liang, S. J. Lin, B. Liu, C. Liu, D. Liu, H. Liu, H. D. Liu, J. Liu, J. L. Liu, J. S. Liu, J. Y. Liu, M. Y. Liu, R. Y. Liu, S. M. Liu, W. Liu, Y. N. Liu, Z. X. Liu, W. J. Long, R. Lu, H. K. Lv, B. Q. Ma, L. L. Ma, X. H. Ma, J. R. Mao, A. Masood, W. Mitthumsiri, T. Montaruli, Y. C. Nan, B. Y. Pang, P. Pattarakijwanich, Z. Y. Pei, M. Y. Qi, B. Q. Qiao, D. Ruffolo, V. Rulev, A. Sáiz, L. Shao, O. Shchegolev, X. D. Sheng, J. R. Shi, H. C. Song, Y. V. Stenkin, V. Stepanov, Q. N. Sun, X. N. Sun, Z. B. Sun, P. H. T. Tam, Z. B. Tang, W. W. Tian, B. D. Wang, C. Wang, H. Wang, H. G. Wang, J. C. Wang, J. S. Wang, L. P. Wang, L. Y. Wang, R. N. Wang, W. Wang, W. Wang, X. G. Wang, X. J. Wang, X. Y. Wang, Y. D. Wang, Y. J. Wang, Y. P. Wang, Z. Wang, Z. Wang, Z. H. Wang, Z. X. Wang, D. M. Wei, J. J. Wei, Y. J. Wei, T. Wen, C. Y. Wu, H. R. Wu, S. Wu, W. X. Wu, X. F. Wu, S. Q. Xi, J. Xia, J. J. Xia, G. M. Xiang, G. Xiao, H. B. Xiao, G. G. Xin, Y. L. Xin, Y. Xing, D. L. Xu, R. X. Xu, L. Xue, D. H. Yan, C. W. Yang, F. F. Yang, J. Y. Yang, L. L. Yang, M. J. Yang, R. Z. Yang, S. B. Yang, Y. H. Yao, Z. G. Yao, Y. M. Ye, L. Q. Yin, N. Yin, X. H. You, Z. Y. You, Y. H. Yu, Q. Yuan, H. D. Zeng, T. X. Zeng, W. Zeng, Z. K. Zeng, M. Zha, X. X. Zhai, B. B. Zhang, H. M. Zhang, H. Y. Zhang, J. L. Zhang, J. W. Zhang, L. Zhang, L. Zhang, L. X. Zhang, P. F. Zhang, P. P. Zhang, R. Zhang, S. R. Zhang, S. S. Zhang, X. Zhang, X. P. Zhang, Y. Zhang, Y. Zhang, Y. F. Zhang, Y. L. Zhang, B. Zhao, J. Zhao, L. Zhao, L. Z. Zhao, S. P. Zhao, F. Zheng, Y. Zheng, B. Zhou, H. Zhou, J. N. Zhou, P. Zhou, R. Zhou, X. X. Zhou, C. G. Zhu, F. R. Zhu, H. Zhu, K. J. Zhu, X. Zuo; The Lhaaso Collaboration, Performance of LHAASO-WCDA and observation of the Crab Nebula as a standard candle. *Chin. Phys. C* **45**, 085002 (2021).

56. R. A. Batista, GRB 221009A: A potential source of ultra-high-energy cosmic rays. *arXiv e-prints*, page arXiv:2210.12855, October 2022.
57. A. Rudolph, M. Petropoulou, W. Winter, Ž. Bošnjak, Multi-messenger model for the prompt emission from GRB 221009A. *Astrophys. J. Lett.*, **944**, L34 (2023).

58. G. Raffelt, L. Stodolsky, Mixing of the photon with low-mass particles. *Phys. Rev. D* **37**, 1237–1249 (1988).
59. A. Fletcher, Magnetic fields in nearby galaxies. *ASP Conf. Ser.* **438**, 197–210 (2011).
60. R. Jansson, G. R. Farrar, A new model of the galactic magnetic field. *Astrophys. J.* **757**, 14 (2012).
61. J. M. Cordes, T. J. W. Lazio, NE2001. 1. A New model for the galactic distribution of free electrons and its fluctuations. 7 2002.
62. J. Biteau, D. A. Williams, The extragalactic background light, the Hubble constant, and anomalies: Conclusions from 20 years of TeV gamma-ray observations. *Astrophys. J.* **812**, 60 (2015).
